# Versatile transformations of hydrocarbons in anaerobic bacteria: substrate ranges and regio- and stereo-chemistry of activation reactions^†^

**DOI:** 10.3389/fmicb.2015.00880

**Published:** 2015-09-07

**Authors:** René Jarling, Simon Kühner, Eline Basílio Janke, Andrea Gruner, Marta Drozdowska, Bernard T. Golding, Ralf Rabus, Heinz Wilkes

**Affiliations:** ^1^Organic Geochemistry, Chemistry of the Earth, Helmholtz Centre Potsdam GFZ German Research Centre for GeosciencesPotsdam, Germany; ^2^Department of Microbiology, Max Planck Institute for Marine MicrobiologyBremen, Germany; ^3^School of Chemistry, Newcastle UniversityNewcastle upon Tyne, UK; ^4^General and Molecular Microbiology, Institute for Chemistry and Biology of the Marine Environment, Carl von Ossietzky UniversityOldenburg, Germany; ^5^Organic Geochemistry, Institute for Chemistry and Biology of the Marine Environment, Carl von Ossietzky UniversityOldenburg, Germany

**Keywords:** anaerobic metabolism, hydrocarbons, activation reactions, *n*-alkylsuccinates, detoxification

## Abstract

Anaerobic metabolism of hydrocarbons proceeds either *via* addition to fumarate or by hydroxylation in various microorganisms, e.g., sulfate-reducing or denitrifying bacteria, which are specialized in utilizing *n*-alkanes or alkylbenzenes as growth substrates. General pathways for carbon assimilation and energy gain have been elucidated for a limited number of possible substrates. In this work the metabolic activity of 11 bacterial strains during anaerobic growth with crude oil was investigated and compared with the metabolite patterns appearing during anaerobic growth with more than 40 different hydrocarbons supplied as binary mixtures. We show that the range of co-metabolically formed alkyl- and arylalkyl-succinates is much broader in *n*-alkane than in alkylbenzene utilizers. The structures and stereochemistry of these products are resolved. Furthermore, we demonstrate that anaerobic hydroxylation of alkylbenzenes does not only occur in denitrifiers but also in sulfate reducers. We propose that these processes play a role in detoxification under conditions of solvent stress. The thermophilic sulfate-reducing strain TD3 is shown to produce *n*-alkylsuccinates, which are suggested not to derive from terminal activation of *n*-alkanes, but rather to represent intermediates of a metabolic pathway short-cutting fumarate regeneration by reverse action of succinate synthase. The outcomes of this study provide a basis for geochemically tracing such processes in natural habitats and contribute to an improved understanding of microbial activity in hydrocarbon-rich anoxic environments.

## Introduction

Complex mixtures of structurally diverse hydrocarbons occur naturally in subsurface habitats such as marine sediments and petroleum reservoirs (Tissot and Welte, 1984). As hydrocarbons are energy-rich organic compounds it is not surprising that a variety of microorganisms capable of utilizing these substrates have evolved (Widdel et al., 2010). During the Anthropocene the excessive technical use of petroleum and its products has resulted in further proliferation of hydrocarbons into the biosphere creating additional carbon and energy sources for such organisms. These have to cope with unfavorable living conditions including limited availability of electron acceptors and nutrients or stress caused by high concentrations of hydrocarbon solvents.

In many hydrocarbon-rich environments anoxic conditions prevail due to the imbalance of electron donor vs. oxygen availability. For more than 25 years, pure cultures of microorganisms have been known which utilize certain hydrocarbons as sole source of carbon and energy under strictly anoxic conditions (Schink, 1985; Lovley et al., 1989; Aeckersberg et al., 1991; Gilewicz et al., 1991). Instead of oxygen these organisms (mainly proteobacteria) use nitrate, ferric iron, or sulfate as electron acceptors. According to their respective hydrocarbon growth substrates these bacteria can be grouped into alkylbenzene or *n*-alkane utilizers. Within these subgroups strain-specific substrate ranges exist (for overviews see Rabus, 2005; Widdel et al., 2010). The majority of the known anaerobic *n*-alkane-utilizing bacteria adds their substrates *via* carbon atom 2 to fumarate, presumably catalyzed by homologs of the radical enzyme (methylalkyl)succinate synthase (Mas) from “*Aromatoleum*” sp. strain HxN1 (Rabus et al., 2001; Grundmann et al., 2008). This reaction yields the corresponding (1-methylalkyl)succinates, which are further metabolized to 4-methylalkanoates *via* CoA-thioester formation, intramolecular rearrangement and decarboxylation (Wilkes et al., 2002). The occurrence of methyl-, ethyl- and propyl-succinates in waters from hot sulfate-reducing environments was reported several times and was linked to the potential activation of the corresponding natural gas hydrocarbons (Duncan et al., 2009; Gieg et al., 2010; Wawrik et al., 2012; Callaghan, 2013). In contrast, alkylsuccinates potentially deriving from higher *n*-alkane homologs have only very rarely been detected (Duncan et al., 2009; Wawrik et al., 2012; Bian et al., 2015). For activation of toluene, alkylbenzene-utilizing strains employ addition to fumarate by benzylsuccinate synthase (Bss) forming 2-benzylsuccinate (Biegert et al., 1996; Leuthner et al., 1998), but further transformation proceeds *via* modified β-oxidation (Leutwein and Heider, 1999). In certain denitrifying bacteria alkylbenzenes may alternatively be activated by dehydrogenases forming the corresponding 1-phenylalkan-1-ols (Rabus and Heider, 1998; Kniemeyer and Heider, 2001a); such enzymatic dehydrogenations are so far unknown from sulfate-reducing bacteria.

By now, among the alkylbenzenes only toluene, ethylbenzene, *o*- and *m*-xylene, and *p*-cymene have been reported to be degraded *via* the corresponding benzylsuccinates in pure cultures of anaerobic bacteria (Biegert et al., 1996; Beller and Spormann, 1997; Krieger et al., 1999; Kniemeyer et al., 2003; Strijkstra et al., 2014). Despite this, Martus and Püttmann (2003) found succinate derivatives originating from C_3+_ alkylbenzenes in water samples from a jet fuel-contaminated aquifer, thus providing evidence for a broader relevance of this activation mechanism. Other studies have shown that several hydrocarbon-utilizing anaerobic bacteria are capable of activating and transforming hydrocarbons they cannot grow with when supplied as pure substrates. This was shown for strain HxN1 which co-activates cycloalkanes (Wilkes et al., 2003) and toluene (Rabus et al., 2011) while growing with *n*-hexane. Co-metabolism of toluene was also found in the denitrifying strain OcN1 and the sulfate-reducing strain TD3 (Rabus et al., 2011). In contrast, such kinds of co-activation, especially of alkanes, have not been observed in alkylbenzene-utilizing bacteria. However, a comprehensive survey of co-metabolic capabilities of anaerobic hydrocarbon-degrading bacteria is as yet lacking.

The alkyl-/arylalkyl-succinates formed as initial products of anaerobic metabolism of hydrocarbons contain at least one stereogenic carbon atom. The activation of toluene by strains T and K172 yields exclusively (*R*)-2-benzylsuccinate proving stereoselective addition to fumarate (Beller and Spormann, 1998; Leutwein and Heider, 1999). During anaerobic growth of strain HxN1 with *n*-hexane a mixture of (2*R*,1′*R*)- and (2*S*,1′*R*)-(1-methylpentyl)succinate is formed (Jarling et al., 2012). It is well-known that enzymatic reactions usually lead to enantiopure products (Alworth, 1972). Therefore, it is unlikely that this finding is due to a relaxed stereoselectivity of the activating enzyme Mas, but rather a consequence of a subsequent epimerase activity generating the required stereochemistry for the following rearrangement reaction.

Here, we present a systematic investigation of the formation of alkyl-/arylalkyl-succinates, their stereochemistry and further metabolic activity in hydrocarbon-utilizing anaerobic bacteria. In particular we attempt a detailed comparison of metabolic capabilities of *n*-alkane- vs. alkylbenzene-utilizing organisms. Moreover, we discuss why enzymes assumed to be Mas-like are potentially polyfunctional.

## Materials and methods

### General information

Chemicals and solvents were obtained from reputable suppliers. Where necessary, solvents were either dried using standard techniques or purchased as anhydrous. All reference standards used for identification of metabolites were either purchased from reputable suppliers or synthesized in this (see below) or in previous studies (Rabus et al., 2001; Wilkes et al., 2003; Kniemeyer et al., 2007; Jarling et al., 2012; Strijkstra et al., 2014). Glassware was dried in an oven prior to use. Column chromatography was carried out using 40–60 μm mesh silica in glass columns under medium pressure. NMR spectra were recorded at room temperature with a Bruker Avance III 300 or a Bruker Avance II 500 (both Bruker, Karlsruhe, Germany) or a Jeol ECS-400 instrument (JEOL USA, Peabody, Massachusetts, USA) at the radiofrequency given below. Signals were assigned using ^1^H, ^13^C, DEPT/APT and H,H-COSY spectra. IR spectra were recorded on a Bio-Rad FTS 3000MX diamond ATR. GC-MS analyses were performed using either a Trace GC-MS system (Thermo Fisher Scientific Inc., Thermo Scientific, Braunschweig, Germany) or an Agilent 6890A gas chromatograph coupled to a MAT95 XL mass spectrometer (Thermo Fisher Scientific Inc., Thermo Scientific, Braunschweig, Germany). The gas chromatograph was equipped with a 5% phenyl polysilphenylene-siloxane fused silica capillary column (BPX-5, SGE; 50 m and 0.22 mm i.d., 0.25 μm film thickness). The column temperature was initially held at 50°C for 1 min, then programmed to 310°C at a rate of 3°C/min with a final hold time of 30 min. Helium was used as the carrier gas. The PTV injector temperature was programmed from 50 to 300°C (10 min isothermal) at a rate of 10°C/sec, and the injection volume was 1 μL in the splitless mode. The mass spectrometer was operated in EI mode (70 eV) at a source temperature of 260°C. Full scan mass spectra were recorded from *m/z* 50 to 600. For analytical data of the synthesized standards see the Supplementary Material.

### Cultivation and extraction

The denitrifying or sulfate-reducing, hydrocarbon-degrading bacteria used in this study are compiled in Table S1 in the Supplementary Material. They have been subcultured in our laboratory since their isolation. General procedures for anaerobic cultivation and preparation of media were as described by Widdel et al. (2010). The anoxic bicarbonate-buffered defined mineral media for sulfate reducers (28 mM sulfate, sulfide-reduced) and denitrifiers (10 mM nitrate, ascorbate-reduced) were composed as described previously (Rabus et al., 1993; Rabus and Widdel, 1995). Anoxic sterile crude oil was prepared and added to the cultures as detailed by Rabus and Widdel (1996). Individual aromatic and/or aliphatic hydrocarbons were provided as dilutions in 2,2,4,4,6,8,8-heptamethylnonane with percentages as listed in the Supplementary Table S2. The individual strains were adapted to anaerobic growth with *n*-hexane (strain HxN1), *n*-octane (strain OcN1), *n*-decane (strain TD3), toluene (strains K172, EbN1, ToN1, T, Tol2), *m*-xylene (strains mXyN1 and mXyS1), or *o*-xylene (strain oXyS1) for at least five passages prior to inoculation of the main cultures (400 mL). Main cultures were run in triplicate. Positive controls contained the adaptation substrate only, while negative controls were devoid of electron acceptor or inoculum. All cultures were incubated at 28°C, except for strain TD3 (60°C; Rueter et al., 1994). Harvest of the main cultures for metabolite extraction (see below) was performed when nitrate/nitrite were depleted from the medium and sulfide formation ceased, respectively.

Metabolite extraction was essentially performed as described (Rabus et al., 2001). After heat inactivation, the culture broth was separated from the carrier phase (in the case of crude oil experiments, the nonpolar fraction was removed by successive extraction with *n*-pentane), acidified with HCl and extracted 3 times with 50 mL diethyl ether. The combined ether extracts were dried over anhydrous sodium sulfate and stored at 4°C in glass bottles sealed with Teflon-coated screw caps. The extracts were divided into two aliquots, each reduced to about 1 mL using a TurboVap® 500 (Biotage, Uppsala, Sweden). One aliquot was subsequently methylated with diazomethane as described elsewhere (Rabus et al., 2001). The other aliquot was used for stereochemical investigation as described below.

### Syntheses of succinate derivatives potentially deriving from butyl- and pentyl-benzene

With one exception (see below), a procedure was adapted from Müller et al. (1999) and Concellon et al. (2004), for the condensation after Stobbe (1899) and the reduction, respectively. The Stobbe condensation of diethyl succinate and suitable ketones yields the 1-ethyl esters of the corresponding itaconates. Taking this into account we started with dimethyl succinate to obtain the corresponding 1-methyl esters of the desired products, which after methylation with diazomethane served as standard compounds for comparison with metabolites detected in methylated extracts of bacterial cultures.

About 1 g (44 mmol) sodium was completely dissolved in 8 mL methanol under reflux. After cooling to room temperature, 1.5 mL (1.7 g, 11.6 mmol) dimethyl succinate and subsequently about 6.5 mmol of the respective ketone were added. The mixture was refluxed for 2 h and left overnight at room temperature. After addition of 25 mL water the mixture was extracted three times with 20 mL chloroform. The organic extracts were discarded and the aqueous phase was acidified to pH < 1 using concentrated hydrochloric acid and extracted three times with 20 mL chloroform and once with 30 mL ethyl acetate. The combined organic extracts were dried over sodium sulfate and evaporated under reduced pressure. The yellow-brown crude product was pre-purified by medium pressure chromatography on silica by gradient elution with methanol in chloroform. The product mixture obtained was pure enough for the next step.

About 50–100 mg of the product from the first step were dissolved in 2 mL tetrahydrofuran and 2 mL water, de-aerated und kept under nitrogen. To this solution 12.5 mL 0.1 M blue samarium diiodide solution in tetrahydrofuran was added and the mixture was stirred under nitrogen for about 90 min until the pinkish color diminished. After addition of 5 mL 0.1 M hydrochloric acid the solution was extracted three times with ethyl acetate. The combined extracts were dried over sodium sulfate and evaporated under reduced pressure. The desired products were isolated from the crude product mixtures using semi-preparative HPLC (Prominence system, Shimadzu, Duisburg, Germany) equipped with an Acclaim® OA column (5 μm, 120 Å, 4.0 × 250 mm; Thermo Fisher Scientific Inc., Thermo Scientific Dionex, Dreieich, Germany) applying gradients of acetonitrile in water (both acidified with 0.1% formic acid).

### Syntheses of dimethyl 2-(1-phenylethyl)succinate

(1-Iodoethyl)benzene (1.00 g, 4.31 mmol), tris(trimethylsilyl)silane (1.34 g, 5.39 mmol), dimethyl fumarate (1.86 g, 12.9 mmol), and azobis-isobutyronitrile (0.5 eq) in α,α,α-trifluorotoluene (40 mL) was heated under reflux for 12 h. The mixture was cooled and concentrated to an oil that was fractionated by medium pressure chromatography, eluting with petrol—ethyl acetate (8: 2, v/v) to afford dimethyl 2-(1-phenylethyl)succinate (0.54 g, 50%), as a colorless oil. *R*_f_ 0.40 (petrol—ethyl acetate, 8: 2, v/v). ^1^H and ^13^C NMR indicated a 1:1 mixture of two diastereoisomers.

### Syntheses of 2-(1-phenylethyl)succinic acid

To dimethyl 2-(1-phenylethyl)succinate (0.70 g, 2.79 mmol) in tetrahydrofuran (55 mL) was added, dropwise with stirring, lithium hydroxide (0.27 g, 11 mmol) in water (20 mL). After stirring overnight at room temperature, the tetrahydrofuran was removed under reduced pressure and the remaining aqueous phase was acidified with 6 M hydrochloric acid. The mixture was extracted with ethyl acetate (3 × 20 mL). The combined organic extracts were dried (Na_2_SO_4_) and the solvent was removed to give the crude 2-alkylsuccinic acid. Crystallization of the crude product from ethyl acetate-petrol by the vapor diffusion technique gave 2-(1-phenylethyl)succinic acid (0.433 g, 70%) as white crystals. ^1^H NMR indicated that the product was a 1:1 mixture of diastereoisomers.

### Succinimide formation from diacid derivatives

The method used here was derived from a method published earlier (Jarling et al., 2012). The culture extract or about 100 μg of the standard compound was introduced into a 10 mL pear-shaped flask. 20 μL (*R*)-1-phenylethanamine in diethyl ether (7% v/v) was added and the mixture was dried under a stream of nitrogen. 1 mL toluene and 20 μL pyridine were added und the mixture was refluxed for 1 h. After cooling, 1 mL 3M hydrochloric acid was added and the organic phase was separated. The remaining acidified aqueous solution was extracted twice with 1 mL diethyl ether. The combined organic phases were dried under reduced pressure at 40°C and re-dissolved in 1 mL dichloromethane for GC-MS measurement.

## Results

### Hydrocarbon substrates for *In vivo* formation of succinate derivatives

**Figure 1:**
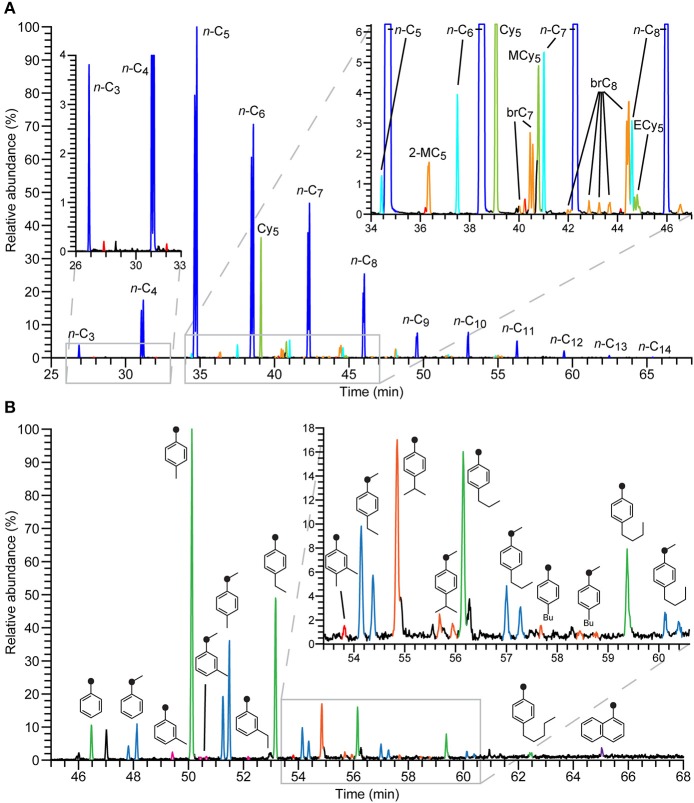
**Partial ion chromatograms (upper trace, *m/z* 114; lower trace, *m/z* 226+236 + 250+264+278+292+306) of a methylated extract of strain TD3 after anaerobic growth with crude oil**. Annotated peaks represent alkyl-/arylalkyl-succinates formed from respective hydrocarbon precursors. **(A)** Homologous series of activation products of *n*-alkanes in blue, branched alkanes in orange and cycloalkanes in green. The red series indicates *n*-alkylsuccinates not directly derived from *n*-alkanes (Annotation code: *n*-C_x_, *n*-alkanes; 2-MC_5_, 2-methylpentane; brC_x_, branched alkanes; Cy_5_, cyclopentane; MCy_5_, methylcyclopentane; ECy_5_, ethylcyclopentane). **(B)** Succinates derived from aromatic hydrocarbons as indicated by their structures. Their respective activation positions are highlighted by black dots. (green, homologous series derived from *p*-*n*-alkyltoluenes; blue, homologous series derived from *p*-*n*-alkylethylbenzenes; magenta, succinates derived from *m*-dialkylbenzenes; orange, succinates derived from *p*-dialkylbenzenes with one branched side chain).

The metabolic potential and activity of alkyl-/arylalkyl-succinate formation was investigated with 11 obligately or facultatively anaerobic bacterial strains (Tables 1, 2, Supplementary Table S1). The analyses revealed a high diversity in succinate derivatives formed, especially in case of *n*-alkane-utilizing strains (Figure 1, Supplementary Table S3). To assign unequivocally the succinate derivatives detected in experiments with crude oil to their respective hydrocarbon-precursors, the same bacterial strains were cultivated with defined binary mixtures of a strain-specific hydrocarbon substrate and a second (mostly non growth supporting) hydrocarbon and comparatively analyzed for metabolites. Succinate derivatives can be identified as dimethyl esters in mass chromatograms due to their specific fragmentation pattern, e.g., *m/z* 114, 146, [M-73] as well as [M-31] and *m/z* 131, 145, [M-145], [M-60] as well as [M] for alkyl- and benzyl-succinates, respectively (for further details see Supplementary Material).

**Table 1:** **Number of gas chromatographically separable alkyl-/arylalkyl-succinates formed from different hydrocarbons by *n*-alkane-utilizing bacteria under anoxic conditions**.^a^

**Substrate**	**Strain**					
	**Electron acceptor**					
	**Cultivation condition^b^**					
	**HxN1^c^**		**OcN1^c^**		**TD3^d^**	
	**${\text{NO}}_{3}^{-}$**		**${\text{NO}}_{3}^{-}$**		**${\text{SO}}_{4}^{2-}$**	
	**B**	**O**	**B**	**O**	**B**	**O**
Methane	0	0	0	0	0	0
Ethane	0	0	0	0	0	0
Propane	1	1	2	1	2	2
*n*-Butane	2	2	2	2	2	2
*n*-Pentane	3	3	3	3	3	3
*n*-Hexane	3	3	3	3	3	3
*n*-Heptane	3	3	3	3	3	3
*n*-Octane	3	3	3	3	3	3
*n*-Nonane	3	3	nt	3	3	3
*n*-Decane	0	0	2	2	2	2
*n*-Undecane	nt	0	2	2	2	2
*n*-Dodecane	nt	0	2	2	2	2
*n*-Tridecane	nt	0	2	1	2	1
*n*-Tetradecane	nt	0	nt	0	nt	1
*n*-Hexadecane	nt	0	0	0	1	0
2-Methylpentane	1	1	1	1	1	1
3-Methylpentane	0	0	0	0	0	0
2,2-Dimethylbutane	0	0	0	0	0	0
2,3-Dimethylbutane	0	0	0	0	0	0
Cyclopentane	1	1	1	1	1	1
Methylcyclopentane	1	1	1	1	1	1
Ethylcyclopentane	nt	4	4	4	4	4
Cyclohexane	1	0	nt	0	nt	0
Toluene	1	1	1	1	1	1
Ethylbenzene	2	2	2	2	2	2
Propylbenzene	3	0	1	0	0	0
Butylbenzene	1	0	1	0	1	0
Pentylbenzene	2	0	2	0	0	0
Hexylbenzene	2	0	2	0	2	0
Heptylbenzene	0	0	2	0	2	0
Octylbenzene	0	0	2	0	2	0
Nonylbenzene	nt	0	2	0	2	0
Decylbenzene	nt	0	2	0	2	0
*o*-Xylene	1	0	1	0	1	0
*o*-Ethyltoluene	nt	0	0	0	0	0
*o*-Isopropyltoluene	nt	0	0	0	0	0
*m*-Xylene	1	0	1	0	1	1
*m*-Ethyltoluene	nt	0	3	0	3	3
*m*-Isopropyltoluene	nt	0	0	0	1	0
*p*-Xylene	1	1	1	1	1	1
*p*-Ethyltoluene	nt	3	3	3	3	3
*p*-Propyltoluene	nt	0	nt	1	nt	1
*p*-Isopropyltoluene	nt	0	1	1	1	1
*p*-Butyltoluene	nt	0	nt	1	nt	1
*p*-*tert*-Butyltoluene	nt	0	1	0	1	0
*p*-Pentyltoluene	nt	0	nt	0	nt	1
*p*-Diethylbenzene	nt	2	nt	1	nt	2
*p*-Ethylpropylbenzene	nt	0	nt	2	nt	2
*p*-Ethylisopropylbenzene	nt	0	nt	1	nt	2
*p*-Butylethylbenzene	nt	0	nt	2	nt	2
1,2,3-Trimethylbenzene	nt	0	0	0	0	0
1,2,4-Trimethylbenzene	nt	0	0	0	1	1
1,3,5-Trimethylbenzene	nt	0	0	0	0	0
1,2,3,5-Tetramethylbenzene	nt	0	0	0	0	0
1,2,4,5-Tetramethylbenzene	nt	0	0	0	0	0
Pentamethylbenzene	nt	0	0	0	0	0
Hexamethylbenzene	nt	0	0	0	0	0
1-Methylnaphthalene	nt	0	nt	0	nt	1
Growth substrate	Co-substrate			No substrate		

a The numbers qualitatively indicate the count of succinates (as dimethyl esters) formed from the respective substrate detected as chromatographically separable peaks during analyses with GC-MS.

b *Anaerobic growth with binary substrate mixtures (B) or crude oil (O); nt, not tested. In the case of the binary substrate mixtures, the hydrocarbons listed in the left column served as co-substrate, with* n*-hexane (HxN1),* n*-octane (OcN1), or* n*-decane (TD3) as the strain-specific main substrates.*

c Ehrenreich et al., 2000.

d Rueter et al., 1994.

**Table 2:** **Number of gas chromatographically separable arylalkyl-succinates formed from different hydrocarbons by alkylbenzene-utilizing bacteria under anoxic conditions**.^a^

**Substrate**	**Strain**															
	**Electron acceptor**															
	**Cultivation condition^b^**															
	**K172^c^**		**EbN1^d^**		**ToN1^d^**		**mXyN1^d^**		**T^e^**		**Tol2^f^**		**oXyS1^g^**		**mXyS1^g^**	
	**${\text{NO}}_{3}^{-}$**		**${\text{NO}}_{3}^{-}$**		**${\text{NO}}_{3}^{-}$**		**${\text{NO}}_{3}^{-}$**		**${\text{NO}}_{3}^{-}$**		**${\text{SO}}_{4}^{2-}$**		**${\text{SO}}_{4}^{2-}$**		**${\text{SO}}_{4}^{2-}$**	
	**B**	**O**	**B**	**O**	**B**	**O**	**B**	**O**	**B**	**O**	**B**	**O**	**B**	**O**	**B**	**O**
*n*-Hexane	0	0	0	0	0	0	0	0	0	0	0	0	0	0	0	0
Cyclohexane	0	0	nt	0	nt	0	nt	0	nt	0	nt	0	nt	0	nt	0
Methylcyclohexane	nt	0	nt	0	nt	0	nt	0	0	0	nt	0	nt	0	nt	0
Toluene	1	1	1	1	1	1	1	1	1	1	1	1	1	0	1	1
Ethylbenzene	nt	0	0	0	nt	0	nt	0	nt	0	nt	0	nt	0	nt	0
Butylbenzene	nt	0	nt	0	0	0	nt	0	nt	0	0	0	nt	0	nt	0
Pentylbenzene	nt	0	nt	0	0	0	nt	0	nt	0	0	0	nt	0	nt	0
*o*-Xylene	nt	1	1	1	nt	1	1	1	1	1	1	1	1	0	1	1
*o*-Ethyltoluene	nt	1	0	1	nt	1	nt	1	nt	1	nt	0	1	0	nt	0
*m*-Xylene	nt	1	1	1	nt	0	1	1	1	1	1	0	0	0	1	1
*m*-Ethyltoluene	nt	0	0	0	nt	0	nt	0	nt	0	nt	0	nt	0	1	1
*m*-Propyltoluene	nt	0	nt	0	nt	0	nt	0	nt	0	nt	0	nt	0	nt	1
*m*-Isopropyltoluene	nt	0	nt	0	nt	0	nt	0	nt	0	nt	0	nt	0	nt	1
*p*-Xylene	nt	0	0	0	nt	1	1	1	1	1	1	0	nt	0	1	1
*p*-Ethyltoluene	nt	0	0	0	nt	0	nt	0	nt	0	nt	0	nt	0	nt	0
1,2,3-Trimethylbenzene	nt	0	nt	0	nt	0	nt	1	nt	1	nt	0	nt	1	nt	1
1,2,4-Trimethylbenzene	nt	1	nt	1	nt	1	nt	3	nt	3	nt	0	1	1	3	3
1,3,5-Trimethylbenzene	nt	0	nt	0	nt	0	nt	1	nt	1	nt	0	nt	0	1	1
1-Methylnaphthalene	nt	0	nt	0	nt	0	nt	0	nt	1	nt	0	nt	0	nt	0
2-Methylnaphthalene	nt	0	nt	0	nt	0	nt	1	nt	1	nt	0	nt	0	nt	1
Growth substrate						Co-substrate							No substrate			

a The numbers qualitatively indicate the count of succinates (as dimethyl esters) formed from the respective substrate detected as chromatographically separable peaks during analyses with GC-MS.

b *Anaerobic growth with binary substrate mixtures (B) or crude oil (O); nt, not tested. In the case of the binary substrate mixtures, the hydrocarbons listed in the left column served as co-substrate, with toluene (K172, EbN1, ToN1, T, Tol2),* m*-xylene (mXyN1, mXyS1), or* o*-xylene (oXyS1) as the strain-specific main-substrates.*

c Anders et al., 1995.

d Rabus and Widdel, 1995.

e Dolfing et al., 1990.

f Rabus et al., 1993.

g Harms et al., 1999.

The hydrocarbons found to be transformed to succinate derivatives by each strain are compiled in Tables 1, 2. It is evident that the range of hydrocarbons transformable by *n*-alkane-utilizing strains markedly exceeds that of the hydrocarbons supporting growth. In contrast, the putative benzylsuccinate synthases in all tested alkylbenzene-utilizing strains are restricted to a rather limited number of alkylbenzenes containing at least one benzylic methyl group. Most notably, these strains are apparently incapable of activating *n*-alkanes by addition to fumarate. In several cases formation of the corresponding succinate derivative was only observed, if the respective hydrocarbon was supplied in high (but sub-lethal) concentration in a binary substrate mixture experiment, while it could not be detected upon growth of the respective strain with crude oil, which contained the same hydrocarbon, however, in significantly lower concentration (Tables 1, 2). In general, the *n*-alkane-utilizing strains also transform *n*-alkanes with chain lengths somewhat longer or shorter than the growth-supporting *n*-alkanes, as well as a restricted range of branched alkanes apparently with low degree of alkyl substitution, certain monocyclic alkanes and various alkylated aromatic hydrocarbons (Table 1, Figure 1). The broadest range of transformable hydrocarbons is observed with strain TD3.

Significant amounts of succinates deriving from activation of cyclopentane and its methyl and ethyl homologs are detected in cultures of *n*-alkane degraders (Figure 1A, light green). In contrast, products of cyclohexane and methylcyclohexane transformation are near detection limit if present at all, even though the cyclohexanes are similar in concentration or even more abundant in the crude oil compared to the cyclopentanes (see for example Dahlgren et al., 1998). Succinate derivatives of branched alkanes appear at similar relative concentrations as those of methyl- and ethyl-cyclopentane (Figure 1A, orange). From the four branched C_6_-alkanes, only 2-methylpentane is activated by all three *n*-alkane-utilizing strains incubated with binary mixtures or crude oil.

Noteworthy, all three *n*-alkane-utilizing strains also transform monoalkylbenzenes with unbranched alkyl chains of different length as well as certain *o*-, *m*-, and *p*-dialkylbenzenes (Table 1). Upon anaerobic growth of these three strains with crude oil containing similar amounts of the xylene isomers, the succinate derivative of the *p*-isomer is always strongly dominating. Similarly, higher abundance of succinates deriving from other *p*-dialkylbenzenes, i.e., *p*-alkylated toluenes and ethylbenzenes, is observed as depicted in Figure 1B. This preferential accumulation of succinates formed from *p*-dialkylbenzenes is further supported by the finding that during anaerobic growth of strain TD3 with a binary mixture of *n*-decane and *m*-cymene (0.5% of the *p*-isomer present as impurity) equal levels of succinates from both cymene isomers are produced.

### Structure elucidation of alkyl-/arylalkyl-succinates and regiochemistry of hydrocarbon activation

**Figure 2:**
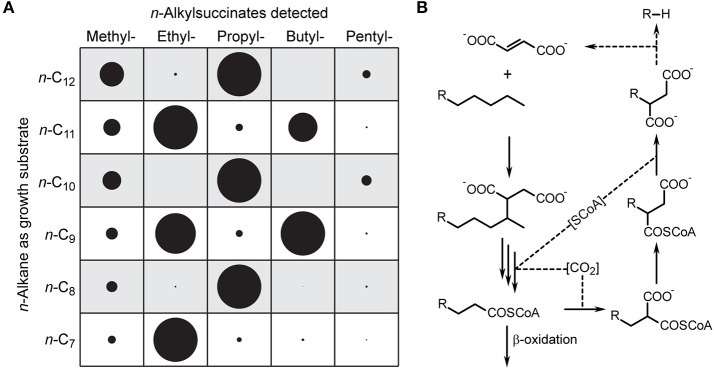
**Formation of short chain *n*-alkylsuccinates from *n*-alkanes with chain length > C_6_ in strain TD3. (A)** Relative amounts of *n*-alkylsuccinates (as dimethyl esters) found in cultures of strain TD3 grown with the mentioned *n*-alkane as sole source of carbon and energy. Relative amounts were determined by integration of respective summed mass traces from GC-MS analyses and normalization to 100% (circle diameter represents the relative amount). **(B)** Potential pathway for formation and further utilization of short chain *n*-alkylsuccinates.

In case of *n*-alkanes it is generally accepted that these molecules are activated at carbon atom 2, leading to a prominent homologous series of (1-methylalkyl)succinate diastereoisomers, when the respective bacteria anaerobically grow with crude oil (Figure 1A, dark blue; see also Wilkes et al., 2003). Only in a few cases activations at the carbon atom 3 (*n*-hexane, strain HxN1) or the terminal methyl group (propane, strain BuS5) have been documented to occur as side reactions (Rabus et al., 2001; Kniemeyer et al., 2007). As confirmed by experiments with single substrates or binary substrate mixtures, all tested *n*-alkane-utilizing strains form succinate derivatives eluting in front of the (1-methylalkyl)succinates (Figure 1A, light blue). These by-products have identical mass spectrometric fragmentation patterns and originate from less favorable transformations of the *n*-alkanes. The respective product from *n*-hexane was identified by comparison with a reference standard as (1-ethylbutyl)succinate in strains OcN1 and TD3 (this study) and HxN1 (Rabus et al., 2001), while tentative assignment of its homologs is based on relative retention times and mass spectrometric fragmentation patterns. Moreover, strains OcN1 and TD3 form isopropyl- as well as *n*-propylsuccinate in cultures with a mixture of their growth substrates (*n*-octane and *n*-decane, respectively) and propane, which were identified by comparisons with reference standards. However, these two strains significantly differ with respect to the ratio of isopropyl- to *n*-propylsuccinate (231.6 in OcN1 vs. 6.5 in TD3). In addition, strain TD3 unexpectedly also formed C_1_–C_7_ *n*-alkylsuccinates during anaerobic growth with pure *n*-alkanes (> C_6_), binary substrate mixtures containing at least one such *n*-alkane or crude oil (Figure 1A, red peaks). Methyl-, *n*-propyl-, and *n*-butylsuccinate were identified by comparison with reference standards, while assignment of the other *n*-alkylsuccinates is based on relative GC retention times and mass spectra. Notably, C-even *n*-alkylsuccinates were predominant upon growth with C-odd *n*-alkanes and *vice versa* (Figure 2A).

The succinates deriving from activation of branched alkanes have not unambiguously been identified due to the lack of standards. We would like to emphasize that only 2-methylpentane, but none of the three other branched C_6_-alkanes, is activated. Compared to the other branched C_6_-alkanes the unique structural feature of 2-methylpentane is an unsubstituted propyl moiety. Thus, we propose that the product of 2-methylpentane should be (1,3-dimethylbutyl)succinate. The regiochemistry of activation of methyl- and ethyl-cyclopentane remains unclear.

To investigate the activation of alkylbenzenes in the three *n*-alkane-utilizing strains, the latter were grown with binary mixtures of a C_1–10_ *n*-alkylbenzene and a growth-supporting *n*-alkane. All of these monoalkylbenzenes were found to be activated, but exclusively the succinate derivatives of toluene and ethylbenzene were detected in cultures grown anaerobically with crude oil, too. By comparison with standards they were identified as benzylsuccinate and the two diastereoisomers of (1-phenylethyl)succinate, respectively, confirming the results of Rabus et al. (2011) for strain HxN1. In case of *n*-propylbenzene strain HxN1 forms 3 activation products. The two metabolites eluting later share the same mass spectrometric fragmentation pattern, i.e., base peak at *m/z* 119 while the one eluting first has a base peak at *m/z* 91. These products were tentatively assigned as the two diastereoisomers of (1-methyl-2-phenylethyl)succinate and (1-phenylpropyl)succinate, respectively. Furthermore, the activation products of *n*-butyl- and *n*-pentyl-benzene from all three *n*-alkane utilizers were identified by comparison with synthetic standards as (1-methyl-3-phenylpropyl)- and (1-methyl-4-phenylbutyl)succinate, respectively (Supplementary Figure S1). The mass spectra of the dimethyl esters of these succinates display *m/z* 91 and [M-64] as most intense signals of typically similar height. In comparison, the methylated (1-phenylalkyl)succinates (potential benzyl position activation products) show a clear predominating base peak at *m/z* 91 (Supplementary Figure S1) and elute significantly earlier. The respective main activation products of hexyl- to decyl-benzene were tentatively identified as (1-methyl-omega-phenylalkyl)succinates based on their relative retention times and homologous fragmentation patterns. In addition to these activation products a second series was detected in extracts from cultures containing pentyl- to decyl-benzene, which always elute shortly before the main products. In analogy to the activation of *n*-alkanes and taking into account their homologous mass spectrometric fragmentation patterns these were tentatively assigned as the (1-ethyl-omega-phenylalkyl)succinates.

It is noteworthy that presumptive Mas-like enzymes apparently activate the methyl or ethyl group in alkyltoluenes and alkylethylbenzenes. The resulting alkyl-substituted benzyl- or (1-phenylethyl)succinates (as dimethyl esters) show distinct mass spectrometric fragmentation patterns. While *m/z* [M-60] is the base peak of the former, the latter exhibit *m/z* [M-145] as the base peak. Moreover, due to the formation of an additional stereogenic center, the products from alkylethylbenzenes always occur as two chromatographically separable diastereoisomers. Taking also into account the above mentioned preferential accumulation of *p*-dialkylbenzene activation products by *n*-alkane-utilizing strains we assign the two homologous series of arylsuccinates formed during anaerobic growth with crude oil as (*p*-*n*-alkylbenzyl)succinates (Figure 1B, green) and [1-(*p*-*n*-alkylphenyl)ethyl]succinates (Figure 1B, blue). This is corroborated by results obtained from experiments with binary mixtures of selected dialkylbenzenes and a growth-supporting *n*-alkane (Table 1). Particularly, (*p*-isopropylbenzyl)succinate the activation product of *p*-cymene was identified by standard comparison. It thus is reasonable to assume that [1-(*p*-isopropylphenyl)ethyl]succinate is formed from *p*-ethylisopropylbenzene (Figure 1B, orange). For *p*-cymene no activation at the isopropyl group was observed.

We suppose that the same is true for activation of *o*- and *m*-dialkylbenzenes, as succinate derivatives formed from them show mass spectrometric fragmentation patterns (as dimethyl esters) very similar to those of the respective *para*-compounds. However, in case of *ortho*-substituted derivatives additional peaks at *m/z* [M-18] and [M-64] were observed, probably due to an *ortho*-effect (Schwarz, 1978). This kind of *ortho*-effect can be used to distinguish the three succinates derived from 1,2,4-trimethylbenzene (Supplementary Figure S2). Among the alkylbenzene utilizers, strain mXyS1 forms all three products, whereas strain oXyS1 produces only the second, and *n*-alkane-utilizing strain TD3, exclusively the third eluting one. The latter (as dimethyl ester) in comparison to the others does not exhibit fragments *m/z* 200 [M-64] and 246 [M-18] in its mass spectrum. Thus, we assign the last eluting isomer as (3,4-dimethylbenzyl)succinate containing no substituent in *ortho*-position to the activated carbon atom. The mass spectra of the other two isomers are rather similar; however, their GC retention times differ significantly. In all other cases of arylalkylsuccinates (as dimethyl esters) the *para*-isomer elutes at a later retention time than the *meta*-isomer. It is therefore very probable, that the earlier eluting one is (2,5-dimethylbenzyl)succinate and the second is (2,4-dimethylbenzyl)succinate.

### Stereochemistry of alkyl-/arylalkyl-succinates

**Figure 3:**
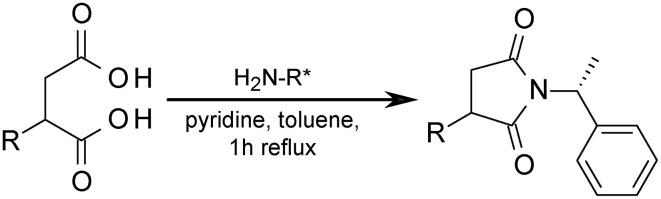
**Formation of diastereoisomeric succinimides from succinate derivatives for stereochemical investigation**. H_2_N-R^*^ = (*R*)-1-phenylethanamine.

The alkyl-/arylalkyl-succinates produced contain at least one stereocenter. For chromatographic separation of the stereoisomers the succinates were transformed to succinimides by reaction with (*R*)-1-phenylethanamine (Figure 3) using a modification of the method described (Jarling et al., 2012). The scope of this derivatisation was evaluated by applying it to a broad range of diacids, including succinic acids with polar and nonpolar substituents as well as malonic and glutaric acids (Supplementary Table S4). Separation efficiency was determined for two achiral GC columns of different polarity. All tested succinic acids with alkyl and (alkyl)benzyl substituents could be separated on both columns under appropriate GC conditions. On the other hand, butanedioic acids with hydroxyl or amino groups (e.g., malic or aspartic acid), as well as malonic or glutaric acids, either gave no imide or the succinimide formed was not separable. Unfortunately, employment of too basic conditions leads to formation of by-products from aryl- and arylalkyl-succinates which might influence the determination of diastereoisomer ratios. The optimized dervatization was then applied to the extracts from the cultures with crude oil, which were analyzed by GC-MS. The succinimides show very characteristic fragmentation patterns allowing assessment of the stereochemistry even in complex mixtures (Supplementary Figure S3, Table S5).

**Figure 4:**
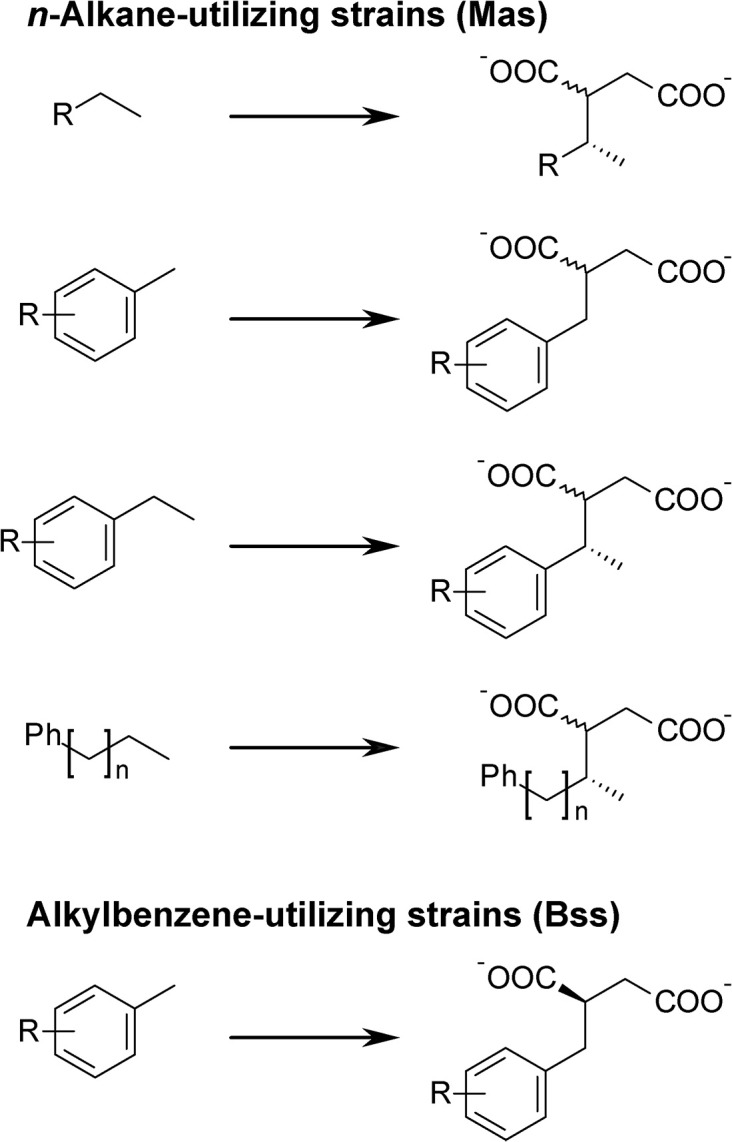
**Stereochemistry of alkyl-/arylalkyl-succinates formed by different strains**. Mas, methylalkylsuccinate synthase; Bss, benzylsuccinate synthase; R = H, alkyl; n = 1…8.

All investigated alkylbenzene-utilizing strains formed exclusively (*R*)-2-benzylsuccinate from toluene, which was confirmed by comparison with a standard. All other succinate derivatives detected in these cultures consisted of exclusively one stereoisomer. As in all cases the later eluting isomer was found, we propose that always the (*R*)-stereoisomer of the succinate is formed (Figure 4). In contrast, the alkyl-/arylalkyl-succinates formed by *n*-alkane utilizers were generally present as two stereoisomers (Figure 4). In case of *n*-hexane-derived (1-methylpentyl)succinate, strains HxN1, OcN1, and TD3 formed the (2*R*,1′*R*)- as well as the (2*S*,1′*R*)-isomer, as confirmed by standard comparison. These are the first and the last eluting of the four possible isomers (Jarling et al., 2012). Likewise, in the cases of (1-phenylethyl)succinate (ethylbenzene-derived) and (1-methyl-4-phenylbutyl)succinate (*n*-pentylbenzene-derived), only the first and last eluting isomers were formed. From equidistant GC retention times we infer that the same is true for all other succinate derivatives with two stereocenters and suggest that they were also present as the epimers at carbon atom 2. Succinate derivatives with only a single stereocenter located in the succinate moiety were always found as mixtures of both enantiomers.

### Further potential activation products from alkylbenzenes

**Figure 5:**
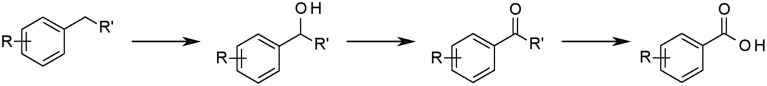
**Anaerobic hydroxylation of aromatic hydrocarbons and further transformations**. R = H, alkyl; R′ = H, methyl, ethyl, propyl.

Unexpectedly, a number of strains produced the corresponding benzyl alcohols and/or 1-phenylalkan-1-ols during anaerobic growth in the presence of certain alkylbenzenes. Furthermore, several strains formed benzaldehydes or 1-phenylalkanones as well as benzoates representing the expected oxidation products of the alcohols (Figure 5, Table 3, Supplementary Table S9). These transformation processes were observed in *n*-alkane- and alkylbenzene-utilizing bacteria and, more importantly, not only in denitrifying but also in sulfate-reducing bacteria. For *n*-alkane-utilizing strains in general, hydroxy- and oxo-derivatives occurred in comparably higher relative abundances in extracts of cultures with *ortho*-di-, tri-, tetra-, penta-, and hexa-alkylbenzenes in comparison with mono- and other dialkylbenzenes. Notably, these appear to be alkylbenzenes that are not or only poorly transformed to succinate derivatives. However, in certain cases succinate derivatives and hydroxylation products originating from the same alkylbenzene were present in the extracts. Respective benzyl alcohols and benzaldehydes were not detected for alkylated toluenes carrying exclusively *meta*-substituents, i.e., *m*-xylene, *m*-ethyltoluene, and 1,3,5-trimethylbenzene. The only exception is tentatively identified *m*-isopropylbenzyl alcohol formed from *m*-cymene in strain TD3.

**Table 3:** **Anaerobic transformation of hydrocarbons by selected bacteria *via* succinate formation or hydroxylation**.^a^

**Hydrocarbon**	**Strain**							
	**Electron acceptor**							
	**Substrate activation^b^**							
	**EbN1**		**mXyS1**		**OcN1**		**TD3**	
	**${\text{NO}}_{3}^{-}$**		**${\text{SO}}_{4}^{2-}$**		**${\text{NO}}_{3}^{-}$**		**${\text{SO}}_{4}^{2-}$**	
	**AS**	**H**	**AS**	**H**	**AS**	**H**	**AS**	**H**
Toluene	+	+	+	−	+	+	+	+
Ethylbenzene	−	+	nt		+	+	+	+
Propylbenzene	nt		nt		−	+	−	+
Butylbenzene	nt		nt		+	+	+	−
*o*-Xylene	+	+	+	−	+	+	+	+
*o*-Ethyltoluene	+	+	nt		−	+	−	+
*o*-Isopropyltoluene	nt		nt		−	+	−	+
*m*-Xylene	+	−	+	−	+	−	+	−
*m*-Ethyltoluene	−	+	+	−	+	+	+	+
*m*-Isopropyltoluene	nt		nt		−	−	+	+
*p*-Xylene	−	+	+	+	+	+	+	+
*p*-Ethyltoluene	−	+	nt		+	+	+	+
*p*-Isopropylbenzene	nt		nt		+	−	+	−
*p*-*tert*-Butylbenzene	nt		nt		+	−	+	−
1,2,3-Trimethylbenzene	nt		nt		−	+	−	+
1,2,4-Trimethylbenzene	nt		+	+	−	+	+	+
1,3,5-Trimethylbenzene	nt		+	−	−	−	−	−
1,2,3,5-Tetramethylbenzene	nt		nt		−	+	−	+
1,2,4,5-Tetramethylbenzene	nt		nt		−	+	−	+
Pentamethylbenzene	nt		nt		−	+	−	+
Hexamethylbenzene	nt		nt		−	+	−	+
Hydroxylation	Both activations				Succinate formation			

a Transformation and further oxidation of the respective hydrocarbon during anaerobic growth of the used bacteria in binary substrate mixture experiments (+, detected; −, not detected; nt, not tested), as revealed by identification of the respective metabolites. For detailed characterization of product formation see Supplementary Table S9.

b Substrate activation via arylalkylsuccinate formation (AS) or anaerobic hydroxylation (H). For details of the cultivation and the strains used see Tables 1, 2.

## Discussion

### Hydrocarbon range, regiochemistry, and stereochemistry

The experiments with crude oil as a complex mixture of hydrocarbons are particularly useful to examine possible preferences for certain hydrocarbon substrates, although it has to be considered that the relative concentrations of metabolites are controlled both by their generation as well as further transformation rates. In case of non-growth supporting co-substrates which are not completely oxidized, the suite of different metabolites formed from each co-substrate represents the actual susceptibility to the enzymes involved. Thus, presumptive Mas-like enzymes seem to prefer as substrates cyclopentane over cyclohexane derivatives, normal over branched alkanes, and *para*- over *ortho*- and *meta*-substituted alkylbenzenes. In contrast, presumptive Bss-like enzymes are restricted to activation of benzylic methyl groups and seem to prefer substrates with low degree of substitution in the aromatic ring. Moreover, all tested alkylbenzene-utilizing strains can activate toluene and *o*-xylene, but further substrates of the respective Bss-like enzymes are specific for each strain (e.g., presumptive Bss from strain oXyS1 requires *ortho*-substituted alkylbenzenes). As these restrictions are likely to be the result of structural differences in the Bss-like enzymes involved, i.e., their amino acid sequences, a phylogenetic analysis would be expected to plot together those enzymes with similar (co-)activation pattern. Indeed, this is the case for BssAs from strains K172 and EbN1, having an identical co-substrate range (Table 2) and giving a distinct branch in the phylogenetic analysis (Strijkstra et al., 2014). In comparison, BssAs from strains T and Tol2, which differ in their (co-)substrate ranges from each other and the former, plot in different, more distant branches. Unfortunately, only very few Bss amino acid sequences are known preventing a detailed comparison of their structure-activity relationship.

In the case of *n*-alkane-utilizing strains the experiments with branched alkanes provide evidence for the necessity of a terminal *n*-propyl group in acyclic aliphatic substrates to enable successful addition to fumarate. In cyclopentane derivatives a ring-CH_2_ group may also act as reactive position, if it is not sterically hindered by neighboring substituents. Rios-Hernandez et al. (2003) provided evidence that carbon atom 3 of ethylcyclopentane is added to fumarate in a sulfate-reducing enrichment culture. Even though the steric constraints at benzylic positions are higher, presumptive Mas-like enzymes seem to be able to activate these positions in toluene, ethylbenzene and their derivatives due to the lower C-H-bond dissociation energy of the benzylic methylene group. In *n*-alkylbenzenes with side chain lengths greater than three, exclusively omega-2 and to a lesser extent omega-3 activation products are formed. Obviously, these substrates are recognized as substituted *n*-alkanes by Mas-like enzymes thus exhibiting the same incomplete regioselectivity as observed for *n*-alkanes (see above). The steric hindrance at the benzylic positions prevents activation at that site, despite the lesser activation energy needed. Steric hindrance might also be the reason why no omega-3 activation by-product of butylbenzene was detected. Propylbenzene is an intermediate case as here the benzylic methylene group is also the omega-3-position; therefore, both activation products were found.

In general the findings from the stereochemical investigations lead to the assumption, that alkyl- as well as arylalkylsuccinates initially formed by *n*-alkane-utilizing strains were further converted, because epimerization should follow CoA-thioester formation. This is further corroborated by the identification of potential downstream products of these succinate derivatives in the respective cultures (see the Supplementary Tables S7, S8 for selected cases).

### *n*-alkylsuccinates in strain TD3 and the possibility of alkanogenesis

The distribution patterns of *n*-alkylsuccinates formed by strain TD3 (i.e., the predominance of C-odd or C-even *n*-alkylsuccinates upon growth with C-even or C-odd *n*-alkanes; Figure 2A, Supplementary Table S6) cannot be explained by assuming a terminal activation of *n*-alkanes as a side-reaction. We rather propose that their formation may be linked to relaxed specificity of the enzymes involved in regeneration of fumarate. Wilkes et al. (2002) had suggested that during anaerobic degradation of *n*-hexane in strain HxN1 propionyl-CoA formed during β-oxidation is used to regenerate fumarate *via* transcarboxylation and intramolecular rearrangement. The degradation of *n*-alkanes leads to *n*-alkanoyl-CoAs, e.g., octanoyl-, hexanoyl-, and butanoyl-CoA are intermediates of *n*-decane degradation. If these *n*-alkanoyl-CoAs instead of propionyl-CoA are transformed by the fumarate-regenerating enzymes, the corresponding *n*-alkylsuccinates will be formed with the observed predominance of odd or even side chains depending on the chain length of the *n*-alkane provided as growth substrate (Figure 2B).

We assume that these metabolites do not reflect “erroneous” side reactions but rather occur in the course of processes that have a specific physiological function. Thus, *n*-alkylsuccinates formed from alkanoyl-CoAs other than propionyl-CoA may serve for alternative fumarate regeneration by reverse action of Mas, which at the same time would result in alkanogenesis. Especially, in case of methylsuccinate (formed from *n*-butanoyl-CoA) methane would be produced. It has been calculated that the overall formation of methylsuccinate from methane and fumarate by Mas is exothermic by 9–11 kcal/mol at room temperature (Beasley and Nanny, 2012). However, we consider that the reverse reaction may take place, if certain thermodynamic conditions favor it: (i) elevated temperature, (ii) very low intracellular concentrations of fumarate, and (iii) removal of methane from the chemical equilibrium out of the cell. Thermophilic strain TD3 compared to mesophilic strains HxN1 and OcN1 (Ehrenreich et al., 2000) grows well at 60°C (Rueter et al., 1994) and solvent stress may lead to fumarate deficiency (see chapter below). It was already shown, that Bss is reversible (Li and Marsh, 2006). Therefore, reversibility of Mas is very likely as well, possibly helping the bacterium to sustain certain combined temperature and solvent stress conditions by avoiding the involvement of the ethylmalonyl-CoA pathway (Erb et al., 2007; Thauer and Shima, 2008) and thus short-cutting the supply of fumarate. Finally, such a pathway may even contribute to biogenic formation of ethane and propane (Hinrichs et al., 2006; Xie et al., 2013) in sediments and possibly also in oil reservoirs.

### Further potential activation products from alkylbenzenes

Based on our observations mentioned above we propose that the respective bacteria listed in Table 3 and Supplementary Table S9 (Supplementary Material) possess enzymes that enable direct oxidation of alkylbenzenes to alcohols. Such activation reactions have previously been reported for anaerobic degradation of ethylbenzene by strain EbN1, propylbenzene by strain PbN1 and *p*-ethyltoluene and *p*-cymene by strain pCyN1 (Rabus and Widdel, 1995; Ball et al., 1996; Johnson and Spormann, 1999; Spormann and Widdel, 2000; Strijkstra et al., 2014). Available evidence suggests that these reactions are catalyzed by dehydrogenases employing molybdenum-containing cofactors and proceed *via* benzylic carbenium ions (Kniemeyer and Heider, 2001a,b; Kloer et al., 2006; Knack et al., 2012). Remarkably, in the case of *p*-ethyltoluene and *p*-cymene activation in strain pCyN1 benzyl alcohols are formed by activation of the methyl groups, which is energetically favorable for *p*- and putatively *o*-substituted dialkylbenzenes due to the hyperconjugative effects of the alkyl substituents stabilizing the intermediary carbenium ion (Strijkstra et al., 2014). This might explain why oxidation products of *meta*-substituted alkylbenzenes were generally not detectable. Interestingly, while such oxidation reactions have so far exclusively been reported from denitrifiers, we here provide evidence that they occur in sulfate-reducing bacteria, too.

### Formation of alkyl-/arylalkyl-succinates and their further transformation as a potential mechanism of hydrocarbon detoxification

Compared with alkylbenzene utilizers, the range of hydrocarbons activated by *n*-alkane utilizers by far exceeds the range of the growth substrates, including the activation of alkylbenzenes, which apparently do not promote growth. In addition, these products of regioselective substrate activation are metabolically further transformed, indicating promiscuity along the downstream degradation steps as well. Webner (2012) showed that a wide range of hydrocarbons, including *n*-alkanes, cyclic alkanes, and alkylbenzenes, induce expression of the *mas* gene in strain HxN1 *in vivo*, and suggested that this may reflect an expanded functionality of this enzyme.

In agreement with this we propose that certain bacteria may benefit from these broad range transformations during detoxification of hydrocarbons. The strong toxic effects of hydrocarbons and other nonpolar solvents on microorganisms and the mechanisms involved in solvent tolerance of bacteria have been investigated intensively (for further information see Sikkema et al., 1995; Kobayashi et al., 2000; Trautwein et al., 2008; Zink and Rabus, 2010; Stancu and Grifoll, 2011; Baumgarten et al., 2012; Segura et al., 2012; Alvarez-Ortega et al., 2013).

We suggest that the alkyl-/arylalkyl-succinate synthase-catalyzed transformation of hydrocarbons to diacids, which under physiological conditions are unable to penetrate the membrane due to their two negative charges, immediately reduces their toxic effects. The further metabolism of these succinates, if formed from substrates not supporting growth may be required for carbon and energy recycling to compensate for the fumarate molecule consumed during the activation. The products of these transformations are monoacids, e.g., benzoate from toluene (Rabus et al., 2011), which may accumulate at the surface of the inner membrane from where they may be extruded. This may explain the typical very low alkyl-/arylalkyl-succinate concentrations in natural environments, in which anaerobic biodegradation of hydrocarbons occurs. Alternatively, if the Mas-mediated reaction is reversible, the intracellular alkyl-/arylalkyl-succinates may be decomposed again, after surrounding concentrations of hydrocarbons have lowered due to external influences. In this case formation of alkyl-/arylalkyl-succinates would work as a fast responding system buffering high intracellular hydrocarbon concentrations and releasing the required fumarate later. If under certain solvent stress conditions intracellular fumarate concentrations are abrogated and potential alternative pathways for regeneration of fumarate are already exploited, the bacteria will still be able to lower their intracellular hydrocarbon concentrations by utilization of the proposed, obviously fumarate-independent oxidation of alkyltoluenes to benzoic acids.

Indeed, a continuous co-transformation of toxic hydrocarbons to their respective succinates and further to monoacids would be expected to result in a substantial accumulation of these compounds in the culture broth which was not observed within the timescales of the cultivation experiments performed in this study. The occurrence of aromatic monoacids in waters from natural hydrocarbon-bearing environments has been reported (e.g., Martus and Püttmann, 2003; Barman Skaare et al., 2007). Here, a massive accumulation might be prevented by the activity of other microorganisms able to further degrade these metabolites and/or under suitable hydrological conditions by water transport processes removing potential dead-end products from the sampling locations. Close to an oil-water contact these amphiphilic acids may also contribute to emulsification of the hydrophobic phase, thus improving the accessibility of the substrates.

## Conclusions

In this study we demonstrate a significantly higher metabolic versatility of *n*-alkane-utilizing anaerobic bacteria with respect to the range of transformable hydrocarbons as compared to known alkylbenzene utilizers. It is thus very likely that an even broader range of nonpolar compounds, which still has to be explored, may represent potential substrates of (methylalkyl)succinate synthases. The requirement for detoxification of hydrocarbons that compromise membranes and do not support growth may offer an explanation for this promiscuity. Conceptually, this would aid especially *n*-alkane-utilizing anaerobic bacteria to survive in environments with relatively high concentrations of complex hydrocarbon mixtures, e.g., oil contaminated aquifers or petroleum reservoirs. The preferential early degradation of *n*-alkanes in oil reservoirs may thus be explained by the more pronounced solvent resistance of *n*-alkane-utilizing anaerobic bacteria which may enable them to survive even in close vicinity to the oil water-contact where their growth substrates are sufficiently bioavailable. Alkylbenzene-utilizing bacteria on the other hand would benefit from the lower oil-water-partition coefficients of their growth substrates (Harms et al., 2010) allowing them to thrive at a greater distance from the oil-water contact, i.e., at lower solvent concentration. The detection of the metabolites identified in this study in natural habitats may be useful for tracing the underlying biochemical processes. However, according to our present results the occurrence of methylsuccinate, particularly if accompanied by other *n*-alkylsuccinates (Gieg et al., 2010; Agrawal and Gieg, 2013), would not necessarily represent an indication of addition of methane to fumarate. Consistently, detection of arylalkylsuccinates together with their respective downstream metabolites (e.g., alkylbenzoic acids) does not necessarily confirm the presence of alkylbenzene-degrading bacteria in the investigated environment (see also Rabus et al., 2011). We point out that the assessment of succinate stereochemistry will help to distinguish the groups of bacteria involved. Furthermore, it may be envisaged that transformation products extruded for the purpose of detoxification may serve as substrates for other members of the microbial community. If this were the case, *n*-alkane-utilizing bacteria may have an elevated role in the subsurface carbon cycle by providing access to inert carbon sources to other kinds of bacteria. In the subsurface microbial community they may take over the part of pioneer organisms by reworking hydrocarbon-infested areas and make them accessible for other community members. Finally, the enormous overall versatility of anaerobic bacteria initiating hydrocarbon metabolism *via* C-H-activation as documented in this study should prompt further investigations on detailed reaction mechanisms, which in the long term may even be relevant with respect to the sustainable use of hydrocarbon feedstocks for the chemical industry.

### Conflict of interest statement

The authors declare that the research was conducted in the absence of any commercial or financial relationships that could be construed as a potential conflict of interest.
