# Supplementary material for: Versatile transformations of hydrocarbons in anaerobic bacteria: substrate ranges and regio- and stereo-chemistry of activation reactions
Source: Front Microbiol. 2015 Sep 7;6:880. doi: 10.3389/fmicb.2015.00880 (PMC4561516; doi:10.3389/fmicb.2015.00880)
Supplement: Supplementary file 1 [file DataSheet1.PDF]

frontiers

Supplementary Material

Versatile transformations of hydrocarbons in anaerobic bacteria: substrate ranges and regio- and stereochemistry of activation reactions

René Jarling[1], Simon Kühner[2], Eline Basílio Janke[1], Andrea Gruner[1], Marta Drozdowska[3], Bernard T. Golding[3], Ralf Rabus[2,4], Heinz Wilkes[1,5,*]

[1]Organic Geochemistry, Helmholtz Centre Potsdam GFZ German Research Centre for Geosciences, Potsdam, Germany
[2]Department of Microbiology, Max Planck Institute for Marine Microbiology, Bremen, Germany
[3]School of Chemistry, Bedson Building, Newcastle University, Newcastle upon Tyne, United Kingdom
[4]General and Molecular Microbiology, Institute for Chemistry and Biology of the Marine Environment (ICBM), Carl von Ossietzky University, Oldenburg, Germany
[5]Organic Geochemistry, Institute for Chemistry and Biology of the Marine Environment (ICBM), Carl von Ossietzky University, Oldenburg, Germany

*** Correspondence:** Heinz Wilkes, Organic Geochemistry, Institute for Chemistry and Biology of the Marine Environment (ICBM), Carl von Ossietzky University, Oldenburg, Germany.
Heinz.wilkes@uni-oldenburg.de

Table of Contents

1. Overview of strains used in this study and their cultivation conditions (Tables S1 & S2)

2. Analytical data of synthetic standards

3. Comparison of mass spectra of synthetic and bacterially formed (phenylalkyl)succinates (Figure S1)

4. Succinates derived from 1,2,4-trimethylbenzene (Figure S2)

5. Succinates detected in cultures upon anaerobic growth (Table S3)

6. Derivatisation of diacids with (*R*)-1-phenylethanamine for stereochemical assignment (Table S4)

7. Stereochemical assignment of succinimides formed from succinates extracted from cultures grown with crude oil (Table S5, Figure S3)

8. *n*-Alkylsuccinates formed by strain TD3 during growth with *n*-alkanes (Table S6)

9. Metabolites of further transformation of selected succinates (Tables S7 & S8)

10. Further activation products of hydrocarbons (Table S9)

11. eiMS data of identified metabolites (Table S10)

1. Overview of strains used in this study

Table S1. Anaerobically hydrocarbon-degrading strains used in this study.

**Strain**	**Hydrocarbon growth substrate(s)**	**Electron acceptor**	**Reference**
**Anaerobic *n*-alkane degraders**			
HxN1	C_5–C_8 *n*-alkanes	Nitrate	Ehrenreich et al. 2000
OcN1	C_8–C_{12} *n*-alkanes	Nitrate	Ehrenreich et al. 2000
TD3	C_6–C_{16} *n*-alkanes	Sulfate	Rueter et al. 1994
**Anaerobic alkylbenzene degraders**			
EbN1	Toluene, ethylbenzene	Nitrate	Rabus et al. 1995
ToN1	Toluene	Nitrate	Rabus et al. 1995
mXyN1	Toluene, *m*-xylene	Nitrate	Rabus et al. 1995
K172	Toluene	Nitrate	Anders et al. 1995
T	Toluene, *m*-xylene	Nitrate	Dolfing et al. 1990
Tol2	Toluene	Sulfate	Rabus et al. 1993
oXyS1	Toluene, *o*-xylene, *o*-ethyltoluene	Sulfate	Harms et al. 1999
mXyS1	Toluene, *m*-xylene, *m*-ethyltoluene	Sulfate	Harms et al. 1999

1. Anders HJ, Kaetzke A, Kämpfer P, Ludwig W, Fuchs G. 1995. Taxonomic position of aromatic-degrading denitrifying pseudomonad strains K 172 and KB 740 and their description as new members of the genera *Thauera*, as *Thauera aromatica* sp. nov., and *Azoarcus*, as *Azoarcus evansii* sp. nov., respectively, members of the beta subclass of the Proteobacteria. Int J SystBacteriol45: 327-33
2. Dolfing J, Zeyer J, Binder-Eicher P, Schwarzenbach RP. 1990. Isolation and characterization of a bacterium that mineralizes toluene in the absence of molecular oxygen. Arch Microbiol 154: 336-41.
3. Ehrenreich P, Behrends A, Harder J, Widdel F. 2000. Anaerobic oxidation of alkanes by newly isolated denitrifying bacteria. Arch Microbiol 173: 58-64.
4. Harms G, Zengler K, Rabus R, Aeckersberg F, Minz D, Rosselló-Mora R, Widdel F. 1999. Anaerobic oxidation of *o*-xylene, *m*-xylene, and homologous alkylbenzenes by new types of sulfate-reducing bacteria. Appl Environ Microbiol 65: 999-1004.
5. Rabus R, Widdel F. 1995. Anaerobic degradation of ethylbenzene and other aromatic hydrocarbons by new denitrifying bacteria. Arch Microbiol 163: 96-103.
6. Rabus R, Nordhaus R, Ludwig W, Widdel F. 1993. Complete oxidation of toluene under strictly anoxic conditions by a new sulfate-reducing bacterium. Appl Environ Microbiol 59: 1444-51.
7. Rueter P, Rabus R, Wilkes H, Aeckersberg F, Rainey FA, Jannasch HW, Widdel F. 1994. Anaerobic oxidation of hydrocarbons in crude oil by new types of sulphate-reducing bacteria. Nature 372: 455-8.

Table S2. Composition of substrates in cultivation experiments with binary mixtures.

Strain[a]
Electron acceptor
Main substrate[b]
Substrate content[c]

	**HxN1**		**OcN1**		**TD3**		**K172**		**EbN1**		**ToN1**		**mXyN1**		**T**		**Tol2**		**oXyS1**		**mXyS1**	
	NO_3^-		NO_3^-		SO_4^{2-}		NO_3^-		NO_3^-		NO_3^-		NO_3^-		NO_3^-		SO_4^{2-}		SO_4^{2-}		SO_4^{2-}	
	***n*-hexane**		***n*-octane**		***n*-decane**		**toluene**		**toluene**		**toluene**		**toluene**		**toluene**		**toluene**		***o*-xylene**		***m*-xylene**	
**(Co-)Substrate**	M	C	M	C	M	C	M	C	M	C	M	C	M	C	M	C	M	C	M	C	M	C
Methane	4%	1atm	4%	1atm	4%	1atm																
Ethane	4%	1atm	4%	1atm	4%	1atm																
Propane	4%	1atm	4%	1atm	4%	1atm																
n-Butane	4%	1atm	4%	1atm	4%	1atm																
n-Pentane	4%	1%	4%	1%	4%	1%																
n-Hexane[e]	4%	-	4%	1%	4%	5%	see below[e]		see below[e]		see below[e]		see below[e]		see below[e]		see below[e]		see below[e]		see below[e]	
n-Heptane	4%	2%	4%	1%	-	5%																
n-Octane	4%	3%	4%	-	-	5%																
n-Nonane	4%	10%			-	5%																
n-Decane	4%	6%	-	5%	2.5%	-																
n-Undecane			-	10%	-	10%																
n-Dodecane			-	10%	-	10%																
n-Tridecane			4%	10%	-	10%																
n-Hexadecane			4%	96%	4%	96%																
2-Methylpentane	4%	1%	4%	1%	4%	1%																
3-Methylpentane	4%	1%	4%	1%	4%	1%																
2,2-Dimethylbutane	4%	4%	4%	4%	4%	4%																
2,3-Dimethylbutane	4%	4%	4%	4%	4%	4%																
Cyclopentane	4%	1%	4%	1%	4%	1%																
Methylcyclopentane	4%	1%	4%	1%	4%	1%																
Ethylcyclopentane			4%	1%	4%	1%																
Cyclohexane	4%	1%					1.5%	0.5%														
Methylcyclohexane															1.5%[f]	0.5%						
Toluene	4%	1%	4%	0.5%	4%	0.5%	1.5%	-	0.5%	-	1.5%	-	1.5%	-	1.5%	-	1.5%	-	1%	1%	1.5%	0.5%
Ethylbenzene	4%	0.5%	4%	0.5%	4%	0.5%			1%	2%												
Propylbenzene	4%	1%	4%	1%	4%	1%																
Butylbenzene	4%	1%	4%	1%	4%	1%					2%	1%					2%	1%				
Pentylbenzene	4%	2%	4%	2%	4%	2%					2%	1%					2%	1%				
Hexylbenzene	4%	5%	4%	5%	4%	5%																
Heptylbenzene	4%	5%	4%	5%	4%	5%																
Octylbenzene	4%	10%	4%	10%	4%	10%																
Nonylbenzene			4%	20%	4%	20%																
Decylbenzene			4%	20%	4%	20%																

Table S2. Continued.

Strain[a]
Electron acceptor
Main substrate[b]
Substrate content[c]

	**HxN1**		**OcN1**		**TD3**		**K172**		**EbN1**		**ToN1**		**mXyN1**		**T**		**Tol2**		**oXyS1**		**mXyS1**	
	NO_3^-		NO_3^-		SO_4^{2-}		NO_3^-		NO_3^-		NO_3^-		NO_3^-		NO_3^-		SO_4^{2-}		SO_4^{2-}		SO_4^{2-}	
	n **-hexane**		***n*** **-octane**		***n*** **-decane**		**toluene**		**toluene**		**toluene**		**toluene**		**toluene**		**toluene**		***o*** **-xylene**		***m*** **-xylene**	
**(Co-)Substrate**	M	C	M	C	M	C	M	C	M	C	M	C	M	C	M	C	M	C	M	C	M	C
o -Xylene	4%	0.5%	4%	0.5%	4%	0.5%			1.5%	0.5%			1.5%	0.5%	1.5%	0.5%	1.5%	0.5%	2%	-	1.5%	0.5%
o -Ethyltoluene			4%	0.5%	4%	0.5%			1.5%	0.5%									1.5%	0.5%		
o -Isopropyltoluene			4%	0.5%	4%	0.5%																
m -Xylene	4%	0.5%	4%	0.5%	4%	0.5%			1.5%	0.5%			-	2%	1.5%	0.5%	1.5%	0.5%	1.5%	0.5%	2%	-
m -Ethyltoluene			4%	0.5%	4%	0.5%			1.5%	0.5%											1.5%	0.5%
m -Isopropyltoluene			4%	0.5%	4%	0.5%																
p -Xylene	4%	0.5%	4%	0.5%	4%	0.5%			1.5%	0.5%			1.5%	0.5%	1.5%	0.5%	1.5%	0.5%	1.5%	0.5%	1.5%	0.5%
p -Ethyltoluene			4%	0.5%	4%	0.5%			2%	1%												
p -Isopropyltoluene			4%	0.5%	4%	0.5%																
p -tert-Butyltoluene			4%	5%	4%	5%																
1,2,3-Trimethylbenzene			4%	1%	4%	1%																
1,2,4-Trimethylbenzene			4%	1%	4%	1%													1.5%	1%	1.5%	1%
1,3,5-Trimethylbenzene			4%	1%	4%	1%															1.5%	1%
1,2,3,5-Tetramethylbenzene			4%	5%	4%	5%																
1,2,4,5-Tetramethylbenzene			4%	5%	4%	5%																
Pentamethylbenzene			4%	10%	4%	10%																
Hexamethylbenzene			4%	10%	4%	10%																

[a]For detailed information on the strains see Tables 1, 2 and S1.
[b]Substrate supporting growth in these experiments
[c]Content (% v/v) of the main substrate (M) and each Co-substrate (C) in 5 mL 2,2,4,4,6,8,8-Heptamethylnonane (HMN), which served as inert carrier phase.
[d]Gaseous hydrocarbons were supplied with the given pressure in the culture head space.
[e]Strains K172, EbN1, ToN1, mXyN1, T, Tol2, oXyS1, mXyS1 were supplied each with the following contents of main substrate + *n* -hexane: 1%+10%, 1%+40%, 0.5%+10%, and 0.5%+40% in HMN.
[f]Here, *m* -xylene served as main substrate.

2. Analytical data of synthetic standards

2-(1-Phenylbutyl)succinic acid 1-methyl ester

Note: Only one of the two diastereoisomers was isolated.

^{1}H NMR (500 MHz, $CDCl_3$, TMS): δ [ppm] 7.23 (ps-t, 2H, J=7.6Hz, *m*-H-Ar), 7.15 (ps-t, 1H, J=7.4Hz, *p*-H-Ar), 7.03 (ps-d, 2H, J=7.6Hz, *o*-H-Ar), 3.66 (s, 3H, $-OCH_3$), 2.93 (ddd, 1H, J_1=11.4Hz, J_2=9.8Hz, J_3=3.5Hz, -OOC-CH-), 2.70 (ddd, 1H, J_1=11.2Hz, J_2=9.8Hz, J_3=4.1Hz, benzyl-CH), 2.51 (dd, 1H, J_1=17.4Hz, J_2=11.4Hz, -OOC-CH_2^A-), 2.13 (dd, 1H, J_1=17.4Hz, J_2=3.5Hz, -OOC-CH_2^B-), 1.63-1.54 (m, 1H, -CH-CH_2^A-CH_2-), 1.49-1.41 (m, 1H, -CH-CH_2^B-CH_2-), 0.98 (sextet, 2H, J=7.3Hz, -CH_2-CH_3), 0.73 (t, 3H, J=7.3, -CH_2-CH_3).

^{13}C NMR (125 MHz, $CDCl_3$, *C*DCl_3): δ [ppm] 176.8 (1C, -COOH), 175.0 (1C, -*C*OO-CH_3), 141.2 (1C, quat. C-Ar), 128.7 (2C, *o*-/*m*-C-Ar), 128.1 (2C, *o*-/*m*-C-Ar), 127.0 (1C, *p*-C-Ar), 51.9 (1C, $-OCH_3$), 47.8 (1C, CH), 47.5 (1C, CH), 36.0 (1C, -*C*H_2-COO-), 34.8 (1C, -CH-*C*H_2-CH_2-), 20.5 (1C, -*C*H_2-CH_3), 13.9 (1C, -CH_2-*C*H_3).

EI-MS (70 eV, as dimethyl ester): *m/z* (rel. int.) 278 (2), 247 (10), 246 (6), 218 (50), 215 (17), 206 (9), 205 (50), 187 (33), 186 (19), 173 (51), 159 (4), 157 (4), 146 (31), 145 (21), 144 (19), 143 (9), 134 (6), 133 (43), 132 (11), 131 (49), 129 (14), 121 (19), 117 (53), 116 (14), 115 (42), 114 (36), 105 (9), 104 (19), 103 (10), 92 (25), 91 (100), 78 (5), 77 (7), 74 (3), 65 (4), 59 (14), 55 (11).

2-(1-Methyl-3-phenylpropyl)succinic acid 1-methyl ester

Note: Both diastereoisomers were isolated in a 2:1 ratio. Data is given for the major diastereoisomer.

^{1}H NMR (500 MHz, acetone-d_6, acetone-d_5): δ [ppm] 7.26 (ps-d, 2H, J=7.5Hz, *o*-H-Ar), 7.22 (ps-t, 2H, J=7.5Hz, *m*-H-Ar), 7.16 (ps-t, 1H, J=7.5Hz, *p*-H-Ar), 3.61 (s, 3H, $-OCH_3$), 2.93-2.86 (m, 1H, -OOC-CH-), 2.73 (dd, 1H, J_1=17.0Hz, J_2=10.8Hz, -OOC-CH_2^A-), 2.74-2.67 (m, 1H, benzyl-CH_2^A), 2.66-2.57 (m, 1H, benzyl-CH_2^B), 2.44 (dd, 1H, J_1=17.0Hz, J_2=4.0Hz, -OOC-CH_2^B-), 1.88-179 (m, 1H, -C*H*-CH_3), 1.77-1.67 (m, 1H, -CH-CH_2^A-CH_2-), 1.55-1.46 (m, 1H, -CH-CH_2^B-CH_2-), 0.98 (d, 3H, J=6.9, -CH-CH_3).

^{13}C NMR (125 MHz, acetone-d_6, CO(*C*D_3)$_2$): δ [ppm] 174.8 (1C, -COOH), 173.6 (1C, -*C*OO-CH_3), 143.2 (1C, quat. C-Ar), 129.19 (2C, *o*-/*m*-C-Ar), 129.16 (2C, *o*-/*m*-C-Ar), 126.5 (1C, *p*-C-Ar), 51.6 (1C, $-OCH_3$), 46.5 (1C, -OOC-*C*H-), 36.9 (1C, -*C*H_2-COO-), 35.4 (1C, -*C*H-CH_3), 34.1 (1C, benzyl-CH_2), 33.7 (1C, -CH-*C*H_2-CH_2-), 16.9 (1C, -CH-*C*H_3).

EI-MS (70 eV, as dimethyl ester): *m/z* (rel. int.) 278 (3), 247 (7), 246 (27), 215 (23), 214 (95), 187 (7), 186 (8), 173 (4), 169 (7), 158 (4), 146 (8), 145 (17), 144 (8), 143 (12), 142 (4), 141 (7), 131 (17), 129 (6), 128 (4), 117 (8), 114 (15), 113 (11), 105 (10), 104 (31), 103 (9), 91 (100), 87 (6), 85 (10), 79 (7), 78 (6), 77 (10), 69 (7), 65 (21), 59 (19), 55 (23).

2-(1-Phenylpentyl)succinic acid 1-methyl ester

Note: Only one of the two diastereoisomers was isolated.

^{1}H NMR (500 MHz, acetone-d_6, acetone-d_5): δ [ppm] 7.34 (ps-t, 2H, J=7.2Hz, *m*-H-Ar), 7.24 (ps-t, 1H, J=7.2Hz, *p*-H-Ar), 7.23 (ps-d, 2H, J=7.0Hz, *o*-H-Ar), 3.66 (s, 3H, $-OCH_3$), 3.01 (ddd, 1H, J_1=11.5Hz, J_2=9.2Hz, J_3=3.4Hz, -OOC-CH-), 2.84-2.78 (m, 1H, benzyl-CH), 2.49 (dd, 1H, J_1=17.0Hz, J_2=11.5Hz, -OOC-CH_2^A-), 2.16 (dd, 1H, J_1=17.0Hz, J_2=3.4Hz, -OOC-CH_2^B-), 1.78-1.68 (m, 1H, -CH-CH_2^A-CH_2-), 1.68-1.59 (m, 1H, -CH-CH_2^B-CH_2-), 1.34-1.14 (m, 2H, -CH_2-CH_2-CH_2-), 1.11-0.96 (m, 2H, -CH_2-CH_3), 0.79 (t, 3H, J=7.4, -CH_2-CH_3).

^{13}C NMR (125 MHz, acetone-d_6, CO(*C*D_3)$_2$): δ [ppm] 175.3 (1C, -COOH), 173.1 (1C, -*C*OO-CH_3), 142.8 (1C, quat. C-Ar), 129.4 (2C, *o*-/*m*-C-Ar), 129.1 (2C, *o*-/*m*-C-Ar), 127.7 (1C, *p*-C-Ar), 51.7 (1C, $-OCH_3$), 48.7 (1C, CH), 48.4 (1C, CH), 35.1 (1C, -*C*H_2-COO-), 34.0 (1C, -CH-*C*H_2-CH_2-), 30.4 (1C, -CH_2-*C*H_2-CH_2-), 23.1 (1C, -*C*H_2-CH_3), 14.2 (1C, -CH_2-*C*H_3).

EI-MS (70 eV, as dimethyl ester): *m/z* (rel. int.) 292 (1), 232 (9), 229 (5), 219 (16), 201 (7), 200 (7), 190 (9), 187 (16), 158 (4), 147 (6), 146 (11), 145 (8), 131 (6), 129 (5), 117 (14), 116 (6), 115 (18), 114 (15), 105 (12), 104 (6), 103 (4), 92 (6), 91 (100), 77 (4).

2-(1-Methyl-4-phenylbutyl)succinic acid 1-methyl ester

Note: Both diastereoisomers were isolated in a 2:1 ratio. Data is given for the major diastereoisomer.

^{1}H NMR (500 MHz, $CDCl_3$, TMS): δ [ppm] 7.21 (ps-t, 2H, J=7.3Hz, *m*-H-Ar), 7.11 (ps-t, 1H, J=7.3Hz, *p*-H-Ar), 7.10 (ps-d, 2H, J=7.3Hz, *o*-H-Ar), 3.60 (s, 3H, $-OCH_3$), 2.80-2.70 (m, 2H, -OOC-CH-&-OOC-CH_2^A-), 2.58-2.45 (m, 2H, benzyl-CH_2), 2.38-2.29 (m, 1H, -OOC-CH_2^B-), 1.81-173 (m, 1H, -*CH*-CH_3), 1.68-1.47 (m, 2H, -CH_2-CH_2-CH_2-), 1.39-1.29 (m, 1H, -CH-CH_2^a-CH_2-), 1.21-1.09 (m, 1H, -CH-CH_2^B-CH_2-), 0.84 (d, 3H, J=6.9, -CH-CH_3).
^{13}C NMR (125 MHz, $CDCl_3$, *C*DCl_3): δ [ppm] 177.2 (1C, -COOH), 174.4 (1C, -*C*OO-CH_3), 142.3 (1C, quat. C-Ar), 128.4 (2C, *o*-/*m*-C-Ar), 128.3 (2C, *o*-/*m*-C-Ar), 125.8 (1C, *p*-C-Ar), 51.7 (1C, $-OCH_3$), 45.7 (1C, -OOC-*C*H-), 35.9 (1C, -CH_2-COO-), 35.0 (1C, -*C*H-CH_3), 33.5 (1C, benzyl-CH_2), 33.0 (1C, -CH_2-CH_2-CH_2-), 29.3 (1C, -CH-CH_2-CH_2-), 16.8 (1C, -CH-CH_3).
EI-MS (70 eV, as dimethyl ester): *m/z* (rel. int.) 292 (5), 261 (11), 260 (19), 229 (14), 228 (87), 227 (8), 226 (5), 201 (10), 186 (7), 183 (6), 173 (4), 159 (4), 157 (4), 146 (11), 145 (17), 144 (36), 143 (14), 141 (18), 129 (20), 117 (13), 115 (6), 114 (21), 113 (15), 109 (4), 105 (10), 104 (23), 103 (7), 99 (4), 92 (11), 91 (100), 90 (14), 87 (9), 81 (13), 79 (8), 78 (7), 77 (11), 74 (5), 69 (8), 67 (8), 65 (21), 59 (21), 55 (33).

Dimethyl 2-(1-phenylethyl)succinate
Note: Mixture of diastereoisomers was isolated.
^{1}H NMR (300 MHz, $CDCl_3$): δ [ppm] 7.36-7.16 (m, 10H, H-Ar), 3.83 (s, 3H, -CH-COO-CH_3), 3.77 (s, 3H, -CH-COO-CH_3), 3.63 (s, 3H, -CH_2-COO-CH_3), 3.59 (s, 3H, -CH_2-COO-CH_3), 3.30-3.20 (m, 1H, CH_3-*CH*-), 3.17-3.10 (m, 1H, CH_3-*CH*-), 3.05-2.86 (m, 2H, -CH-COO-), 2.76 (dd, 1H, J_1=16.8Hz, J_2=10.9Hz, -CH_2^A-COO-), 2.61 (dd, 1H, J_1=16.9Hz, J_2=10.8Hz, -CH_2^A-COO-), 2.36 (dd, 1H, J_1=16.8Hz, J_2=3.7Hz, -CH_2^B-COO-), 2.21 (dd, 1H, J_1=16.9Hz, J_2=3.6Hz, -CH_2^B-COO-), 1.30 (d, 3H, J=6.9Hz, CH_3-CH-), 1.29 (d, 3H, J=6.9Hz, CH_3-CH-).
^{13}C NMR (75 MHz, $CDCl_3$): δ [ppm] 174.8, 174.0, 172.4, 172.1, 143.5, 143.2, 128.6, 128.3, 127.4, 127.4, 126.8, 126.7, 51.5, 51.4, 48.4, 48.2, 42.4, 41.9, 35.3, 32.6, 20.1, 16.6.
IR (cm^{-1}): 2952, 1731, 1435, 1255, 1196, 1162, 1014, 768, 701.
HRMS calcd. for $C_{14}H_{19}O_4$ $[M+H]^+$: *m/z* 251.1278, found: *m/z* 251.1281.

2-(1-Phenylethyl)succinic acid
Note: Mixture of diastereoisomers was isolated.
^{1}H NMR (400 MHz, MeOD): δ [ppm] 7.30-7.15 (m, 10H, H-Ar), 4.88 (s[br], 4H, -COOH), 3.24-3.17 (m, 1H, CH_3-*CH*-), 3.05-3.00 (m, 1H, CH_3-*CH*-), 2.92-2.83 (m, 2H, -CH-COO-), 2.59 (dd, 1H, J_1=16.8Hz, J_2=10.8Hz, -CH_2^A-COO-), 2.44 (dd, 1H, J_1=16.8Hz, J_2=10.8Hz, -CH_2^A-COO-), 2.25 (dd, 1H, J_1=16.8Hz, J_2=3.6Hz, -CH_2^B-COO-), 2.09 (dd, 1H, J_1=16.8Hz, J_2=3.6Hz, -CH_2^B-COO-), 1.30 (d, 3H, J=6.9Hz, CH_3-CH-), 1.27 (d, 3H, J=6.9Hz, CH_3-CH-).
^{13}C NMR (100 MHz, MeOD): δ [ppm] 176.9, 176.2, 174.6, 174.2, 143.8, 143.5, 128.4, 128.1, 127.3, 127.2, 126.6, 126.4, 42.1, 40.7, 34.8, 32.0, 19.4, 15.6.
IR (cm^{-1}): 3400-2400, 1699, 1434, 1391, 1342, 1279, 1243, 1186, 925, 892, 767, 703.
HRMS calcd. for $C_{12}H_{13}O_4$ $[M-H]^-$: *m/z* 221.0818, found: *m/z* 221.0819.

3. Comparison of mass spectra of synthetic and bacterially formed (phenylalkyl)succinates

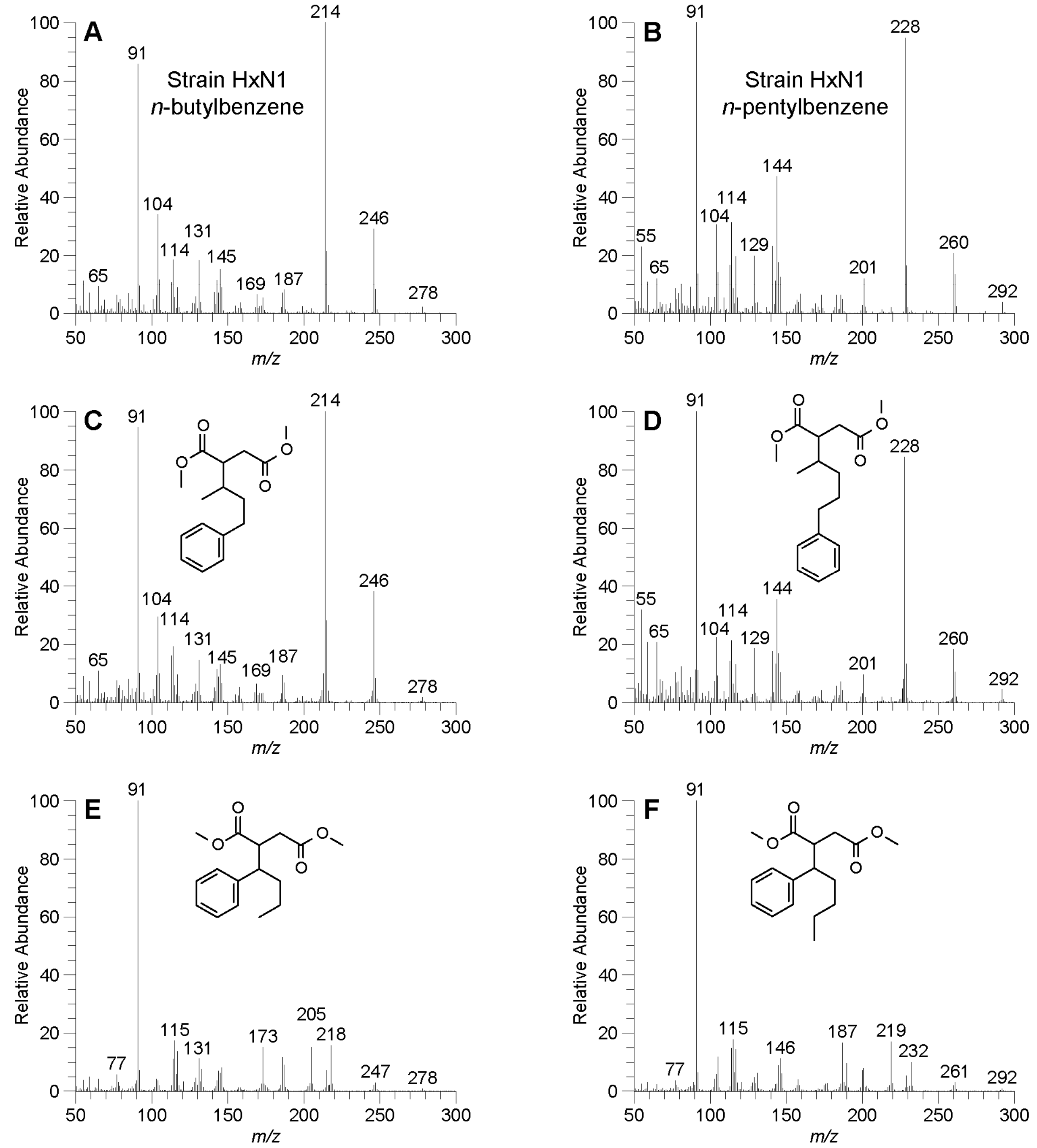

Figure S1. Comparison of electron impact mass spectra of different (phenylalkyl)succinates as dimethyl esters. A) Metabolite formed from *n*-butylbenzene by strain HxN1 upon anaerobic growth. B) Metabolite formed from *n*-pentylbenzene by strain HxN1 upon anaerobic growth. C, D, E, and F) Synthesized standard compounds.

4. Succinates derived from 1,2,4-trimethylbenzene

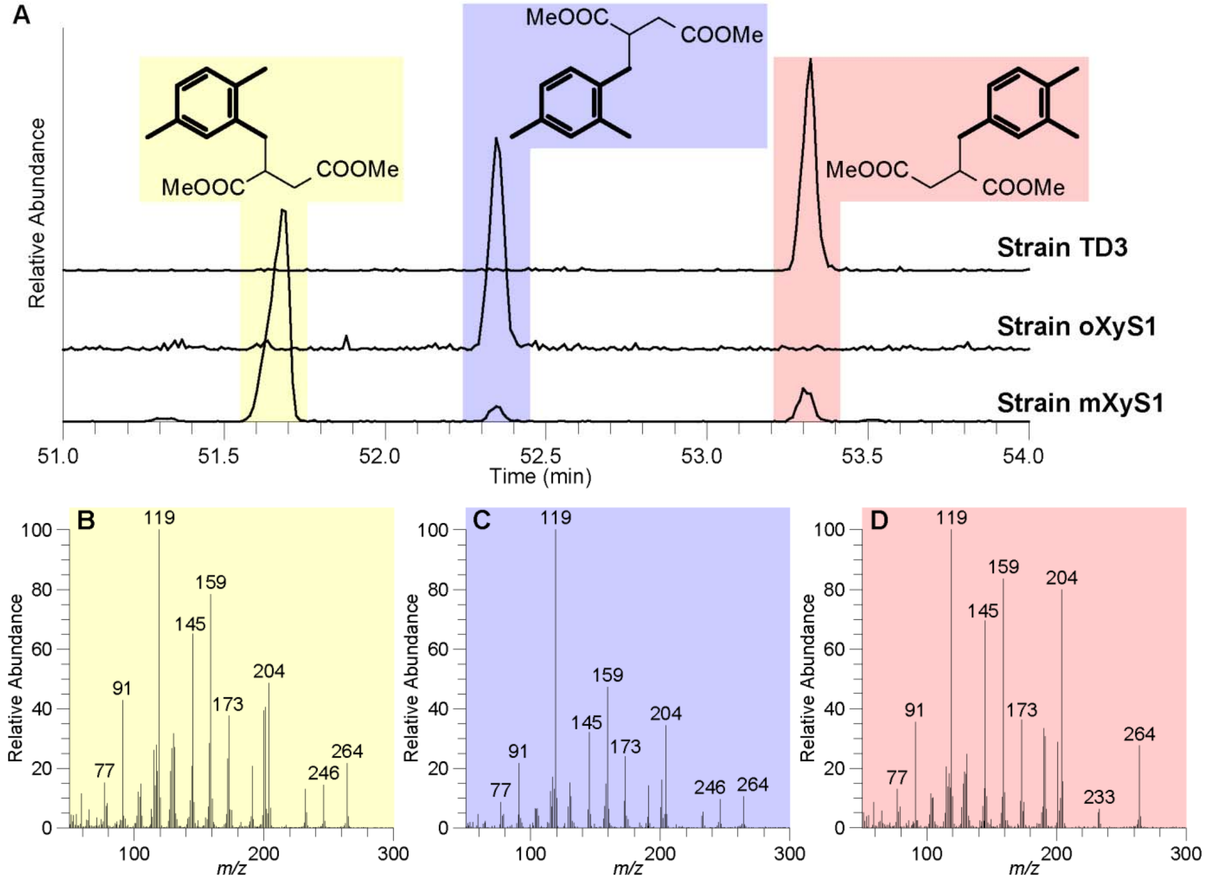

Figure S2. Formation of tentatively identified activation products from 1,2,4-trimethylbenzene in different strains. A) Partial ion chromatograms (*m/z* 264+204) of extracts from cultures of strains TD3 (upper trace), oXyS1 (middle trace) and mXyS1 (lower trace) with their respective growth substrates (*n*-decane, *o*-xylene and *m*-xylene, respectively) amended with 1,2,4-trimethylbenzene. B, C and D) Mass spectra of tentatively identified (2,5-dimethylbenzyl)-, (2,4-dimethylbenzyl)- and (3,4-dimethylbenzyl)succinate.

5. Succinates detected in cultures upon anaerobic growth

Table S3. Succinates detected in cultures upon anaerobic growth with binary mixtures (B) or with crude oil (O).

		**Strain[a]** / Electron acceptor / **Cultivation conditions[b]**																					
		**HxN1**		**OcN1**		**TD3**		**K172**		**EbN1**		**ToN1**		**mXyN1**		**T**		**Tol2**		**oXyS1**		**mXyS1**	
		NO_3^-		NO_3^-		SO_4^{2-}		NO_3^-		NO_3^-		NO_3^-		NO_3^-		NO_3^-		SO_4^{2-}		SO_4^{2-}		SO_4^{2-}	
**Succinate[c]**	**Precursor**	**B**	**O**	**B**	**O**	**B**	**O**	**B**	**O**	**B**	**O**	**B**	**O**	**B**	**O**	**B**	**O**	**B**	**O**	**B**	**O**	**B**	**O**
Isopropylsuccinate	Propane	+	+	+	+	+	+		-		-		-		-		-		-		-		-
n -Propylsuccinate	Propane	-	-	+	-	+	+		-		-		-		-		-		-		-		-
(1-Methylpropyl)succinate (I)	*n* -Butane	+	+	+	+	+	+		-		-		-		-		-		-		-		-
(1-Methylpropyl)succinate (II)	*n* -Butane	+	+	+	+	+	+		-		-		-		-		-		-		-		-
(1-Ethylpropyl)succinate	*n* -Pentane	+	+	+	+	+	+		-		-		-		-		-		-		-		-
(1-Methylbutyl)succinate (I)	*n* -Pentane	+	+	+	+	+	+		-		-		-		-		-		-		-		-
(1-Methylbutyl)succinate (II)	*n* -Pentane	+	+	+	+	+	+		-		-		-		-		-		-		-		-
(1-Ethylbutyl)succinate	*n* -Hexane	+	+	+	+	+	+	-	-	-	-	-	-	-	-	-	-	-	-	-	-	-	-
(1-Methylpentyl)succinate (I)	*n* -Hexane	+	+	+	+	+	+	-	-	-	-	-	-	-	-	-	-	-	-	-	-	-	-
(1-Methylpentyl)succinate (II)	*n* -Hexane	+	+	+	+	+	+	-	-	-	-	-	-	-	-	-	-	-	-	-	-	-	-
(1-Ethylpentyl)succinate	*n* -Heptane	+	+	+	+	+	+		-		-		-		-		-		-		-		-
(1-Methylhexyl)succinate (I)	*n* -Heptane	+	+	+	+	+	+		-		-		-		-		-		-		-		-
(1-Methylhexyl)succinate (II)	*n* -Heptane	+	+	+	+	+	+		-		-		-		-		-		-		-		-
(1-Ethylhexyl)succinate	*n* -Octane	+	+	+	+	+	+		-		-		-		-		-		-		-		-
(1-Methylheptyl)succinate (I)	*n* -Octane	+	+	+	+	+	+		-		-		-		-		-		-		-		-
(1-Methylheptyl)succinate (II)	*n* -Octane	+	+	+	+	+	+		-		-		-		-		-		-		-		-
(1-Ethylheptyl)succinate	*n* -Nonane	+	+		+	+	+		-		-		-		-		-		-		-		-
(1-Methyloctyl)succinate (I)	*n* -Nonane	+	+		+	+	+		-		-		-		-		-		-		-		-
(1-Methyloctyl)succinate (II)	*n* -Nonane	+	+		+	+	+		-		-		-		-		-		-		-		-
(1-Ethyloctyl)succinate	*n* -Decane	-	-	+	+	+	+		-		-		-		-		-		-		-		-
(1-Methylnonyl)succinate	*n* -Decane	-	-	+	+	+	+		-		-		-		-		-		-		-		-
(1-Ethylnonyl)succinate	*n* -Undecane		-	+	+	+	+		-		-		-		-		-		-		-		-
(1-Methyldecyl)succinate	*n* -Undecane		-	+	+	+	+		-		-		-		-		-		-		-		-
(1-Ethyldecyl)succinate	*n* -Dodecane		-	+	+	+	+		-		-		-		-		-		-		-		-
(1-Methylundecyl)succinate	*n* -Dodecane		-	+	+	+	+		-		-		-		-		-		-		-		-
(1-Ethylundecyl)succinate	*n* -Tridecane		-	+	-	+	-		-		-		-		-		-		-		-		-
(1-Methyldodecyl)succinate	*n* -Tridecane		-	+	+	+	+		-		-		-		-		-		-		-		-
(1-Methyltridecyl)succinate	*n* -Tetradecan		-		-		+		-		-		-		-		-		-		-		-
(1-Methylpentadecyl)succinate	*n* -Hexadecane		-	-	-	+	-		-		-		-		-		-		-		-		-

Table S3. Continued.

		Strain[a] / Electron acceptor / Cultivation conditions[b]																					
		HxN1		OcN1		TD3		K172		EbN1		ToN1		mXyN1		T		Tol2		oXyS1		mXyS1	
		NO_3^-		NO_3^-		SO_4^{2-}		NO_3^-		NO_3^-		NO_3^-		NO_3^-		NO_3^-		SO_4^{2-}		SO_4^{2-}		SO_4^{2-}	
**Succinate[c]**	**Precursor**	**B**	**O**	**B**	**O**	**B**	**O**	**B**	**O**	**B**	**O**	**B**	**O**	**B**	**O**	**B**	**O**	**B**	**O**	**B**	**O**	**B**	**O**
(1,3-Dimethylbutyl)succinate	2-Methylpentane	+	+	+	+	+	+		-		-		-		-		-		-		-		-
unidentified isomer	br-C_7		-		-		+		-		-		-		-		-		-		-		-
likely (1,4-Dimethylpentyl)succinate (I)	2-Methylhexane		+		+		+		-		-		-		-		-		-		-		-
likely (1,4-Dimethylpentyl)succinate (II)	2-Methylhexane		+		+		+		-		-		-		-		-		-		-		-
unidentified isomer 1	br-C_8		-		-		+		-		-		-		-		-		-		-		-
unidentified isomer 2	br-C_8		-		-		+		-		-		-		-		-		-		-		-
unidentified isomer 3	br-C_8		-		-		+		-		-		-		-		-		-		-		-
unidentified isomer 4	br-C_8		-		-		+		-		-		-		-		-		-		-		-
likely (1,5-Dimethylhexyl)succinate (I)	2-Methylheptane		-		+		+		-		-		-		-		-		-		-		-
likely (1,5-Dimethylhexyl)succinate (II)	2-Methylheptane		-		+		+		-		-		-		-		-		-		-		-
unidentified isomer	br-C_9		-		-		+		-		-		-		-		-		-		-		-
Cyclopentylsuccinate	Cyclopentane	+	+	+	+	+	+		-		-		-		-		-		-		-		-
(Methylcyclopentyl)succinate	Methylcyclopentane	+	+	+	+	+	+		-		-		-		-		-		-		-		-
unidentified isomer 1	Ethylcyclopentane		+	+	+	+	+		-		-		-		-		-		-		-		-
unidentified isomer 2	Ethylcyclopentane		+	+	+	+	+		-		-		-		-		-		-		-		-
unidentified isomer 3	Ethylcyclopentane		+	+	+	+	+		-		-		-		-		-		-		-		-
unidentified isomer 4	Ethylcyclopentane		+	+	+	+	+		-		-		-		-		-		-		-		-
Cyclohexylsuccinate	Cyclohexane	+	-		-		-		-		-		-		-		-		-		-		-
Benzylsuccinate	Toluene	+	+	+	+	+	+	+	+	+	+	+	+	+	+	+	+	+	+	+	-	+	+
(1-Phenylethyl)succinate (I)	Ethylbenzene	+	+	+	+	+	+		-	-	-		-		-		-		-		-		-
(1-Phenylethyl)succinate (II)	Ethylbenzene	+	+	+	+	+	+		-	-	-		-		-		-		-		-		-
(1-Phenylpropyl)succinate	Propylbenzene	+	-	-	-	-	-		-		-		-		-		-		-		-		-
(1-Methyl-2-phenylethyl)succinate (I)	Propylbenzene	+	-	-	-	-	-		-		-		-		-		-		-		-		-
(1-Methyl-2-phenylethyl)succinate (II)	Propylbenzene	+	-	-	-	-	-		-		-		-		-		-		-		-		-
(1-Methyl-3-phenylpropyl)succinate	Butylbenzene	+	-	+	-	+	-		-		-	-	-		-		-	-	-		-		-
(1-Ethyl-3-phenylpropyl)succinate	Pentylbenzene	+	-	+	-	-	-		-		-	-	-		-		-	-	-		-		-
(1-Methyl-4-phenylbutyl)succinate	Pentylbenzene	+	-	+	-	-	-		-		-	-	-		-		-	-	-		-		-
(1-Ethyl-4-phenylbutyl)succinate	Hexylbenzene	+	-	+	-	+	-		-		-		-		-		-		-		-		-
(1-Methyl-5-phenylpentyl)succinate	Hexylbenzene	+	-	+	-	+	-		-		-		-		-		-		-		-		-
(1-Ethyl-5-phenylpentyl)succinate	Heptylbenzene	-	-	+	-	+	-		-		-		-		-		-		-		-		-
(1-Methyl-6-phenylhexyl)succinate	Heptylbenzene	-	-	+	-	+	-		-		-		-		-		-		-		-		-
(1-Ethyl-6-phenylhexyl)succinate	Octylbenzene	-	-	+	-	+	-		-		-		-		-		-		-		-		-
(1-Methyl-7-phenylheptyl)succinate	Octylbenzene	-	-	+	-	+	-		-		-		-		-		-		-		-		-

Table S3. Continued.

		Strain[a] / Electron acceptor / Cultivation conditions[b]																					
		HxN1		OcN1		TD3		K172		EbN1		ToN1		mXyN1		T		Tol2		oXyS1		mXyS1	
		NO_3^-		NO_3^-		SO_4^{2-}		NO_3^-		NO_3^-		NO_3^-		NO_3^-		NO_3^-		SO_4^{2-}		SO_4^{2-}		SO_4^{2-}	
**Succinate**[c]	**Precursor**	**B**	**O**	**B**	**O**	**B**	**O**	**B**	**O**	**B**	**O**	**B**	**O**	**B**	**O**	**B**	**O**	**B**	**O**	**B**	**O**	**B**	**O**
(1-Ethyl-7-phenylheptyl)succinate	Nonylbenzene		-	+	-	+	-		-		-		-		-		-		-		-		-
(1-Methyl-8-phenyloctyl)succinate	Nonylbenzene		-	+	-	+	-		-		-		-		-		-		-		-		-
(1-Ethyl-8-phenyloctyl)succinate	Decylbenzene		-	+	-	+	-		-		-		-		-		-		-		-		-
(1-Methyl-9-phenylnonyl)succinate	Decylbenzene		-	+	-	+	-		-		-		-		-		-		-		-		-
(2-Methylbenzyl)succinate	*o*-Xylene	+	-	+	-	+	-		+	+	+		+	+	+	+	+	+	+	+	-	+	+
(2-Ethylbenzyl)succinate	*o*-Ethyltoluene		-	-	-	-	-		+	-	+		+		+		+		-	+	-		-
(3-Methylbenzyl)succinate	*m*-Xylene	+	-	+	-	+	+		+	+	+		-	+	+	+	+	+	-	-	-	+	+
[1-(3-Methylphenyl)ethyl]succinate (I)	*m*-Ethyltoluene		-	+	-	+	+		-	-	-		-		-		-		-		-	-	-
[1-(3-Methylphenyl)ethyl]succinate (II)	*m*-Ethyltoluene		-	+	-	+	+		-	-	-		-		-		-		-		-	-	-
(3-Ethylbenzyl)succinate	*m*-Ethyltoluene		-	+	-	+	+		-	-	-		-		-		-		-		-	+	+
(3-Isopropylbenzyl)succinate	*m*-Isopropyltoluene		-	-	-	+	-		-		-		-		-		-		-		-		+
(3-Propylbenzyl)succinate	*m*-Propyltoluene		-		-		-		-		-		-		-		-		-		-		+
(3-C_4-benzyl)succinate	*m*-(*n*-, *sec*- or *iso*-)Butyltoluene		-		-		-		-		-		-		-		-		-		-		+
(3-C_4-benzyl)succinate	*m*-(*n*-, *sec*- or *iso*-)Butyltoluene		-		-		-		-		-		-		-		-		-		-		+
(4-Methylbenzyl)succinate	*p*-Xylene	+	+	+	+	+	+		-	-	-		+	+	+	+	+	+	-		-	+	+
[1-(4-Methylphenyl)ethyl]succinate (I)	*p*-Ethyltoluene		+	+	+	+	+		-	-	-		-		-		-		-		-		-
[1-(4-Methylphenyl)ethyl]succinate (II)	*p*-Ethyltoluene		+	+	+	+	+		-	-	-		-		-		-		-		-		-
(4-Ethylbenzyl)succinate	*p*-Ethyltoluene		+	+	+	+	+		-	-	-		-		-		-		-		-		-
[1-(4-Ethylphenyl)ethyl]succinate (I)	*p*-Diethylbenzene		+		+		+		-		-		-		-		-		-		-		-
[1-(4-Ethylphenyl)ethyl]succinate (II)	*p*-Diethylbenzene		+		+		+		-		-		-		-		-		-		-		-
(4-Isopropylbenzyl)succinate	*p*-Isopropyltoluene		-	+	+	+	+		-		-		-		-		-		-		-		-
(4-Propylbenzyl)succinate	*p*-Propyltoluene		-		+		+		-		-		-		-		-		-		-		-
[1-(4-Isopropylphenyl)ethyl]succinate (I)	*p*-Ethylisopropylbenzene		-		+		+		-		-		-		-		-		-		-		-
[1-(4-Isopropylphenyl)ethyl]succinate (II)	*p*-Ethylisopropylbenzene		-		+		+		-		-		-		-		-		-		-		-
[1-(4-Propylphenyl)ethyl]succinate (I)	*p*-Ethylpropylbenzene		-		+		+		-		-		-		-		-		-		-		-
[1-(4-Propylphenyl)ethyl]succinate (II)	*p*-Ethylpropylbenzene		-		+		+		-		-		-		-		-		-		-		-
(4-*tert*-Butylbenzyl)succinate	*p*-*tert*-Butyltoluene		-	+	-	+	-		-		-		-		-		-		-		-		-
(4-C_4-benzyl)succinate	*p*-(*sec*- or *iso*-)Butyltoluene		-		+		+		-		-		-		-		-		-		-		-
[1-(4-C_4-phenyl)ethyl]succinate (I)	*p*-(*sec*- or *iso*-)Butylethylbenzene		-		+		+		-		-		-		-		-		-		-		-
[1-(4-C_4-phenyl)ethyl]succinate (II)	*p*-(*sec*- or *iso*-)Butylethylbenzene		-		+		+		-		-		-		-		-		-		-		-
(4-Butylbenzyl)succinate	*p*-Butyltoluene		-		+		+		-		-		-		-		-		-		-		-
[1-(4-Butylphenyl)ethyl]succinate (I)	*p*-Butylethylbenzene		-		+		+		-		-		-		-		-		-		-		-
[1-(4-Butylphenyl)ethyl]succinate (II)	*p*-Butylethylbenzene		-		+		+		-		-		-		-		-		-		-		-
(4-Pentylbenzyl)succinate	*p*-Pentyltoluene		-		-		+		-		-		-		-		-		-		-		-

Table S3. Continued.

Succinate[c]	Precursor	HxN1 (NO_3^-) B	HxN1 (NO_3^-) O	OcN1 (NO_3^-) B	OcN1 (NO_3^-) O	TD3 (SO_4^{2-}) B	TD3 (SO_4^{2-}) O	K172 (NO_3^-) B	K172 (NO_3^-) O	EbN1 (NO_3^-) B	EbN1 (NO_3^-) O	ToN1 (NO_3^-) B	ToN1 (NO_3^-) O	mXyN1 (NO_3^-) B	mXyN1 (NO_3^-) O	T (NO_3^-) B	T (NO_3^-) O	Tol2 (SO_4^{2-}) B	Tol2 (SO_4^{2-}) O	oXyS1 (SO_4^{2-}) B	oXyS1 (SO_4^{2-}) O	mXyS1 (SO_4^{2-}) B	mXyS1 (SO_4^{2-}) O
		**Strain[a]** / Electron acceptor / **Cultivation conditions[b]**																					
(2,6-Dimethylbenzyl)succinate	1,2,3-Trimethybenzene		-	-	-	-	-		-		-		-		-		-		-		+		-
(2,3-Dimethylbenzyl)succinate	1,2,3-Trimethybenzene		-	-	-	-	-		-		-		-		+		+		-		-		+
(2,5-Dimethylbenzyl)succinate	1,2,4-Trimethylbenzene		-	-	-	-	-		+		+		+		+		+		-	-	-	+	+
(2,4-Dimethylbenzyl)succinate	1,2,4-Trimethylbenzene		-	-	-	-	-		-		-		-		+		+		-	+	+	+	+
(3,4-Dimethylbenzyl)succinate	1,2,4-Trimethylbenzene		-	-	-	+	+		-		-		-		+		+		-	-	-	+	+
(3,5-Dimethylbenzyl)succinate	1,3,5-Trimethylbenzene		-	-	-	-	-		-		-		-		+		+		-		-	+	+
(Naphth-1-ylmethyl)succinate	1-Methylnaphthalene		-		-		+		-		-		-		-		+		-		-		-
(Naphth-2-ylmethyl)succinate	2-Methylnaphthalene		-		-		-		-		-		-		+		+		-		-		+

Detected (+) | Not tested | Not detected (-)

[a]For detailed information on the strains and their specific main substrates see Tables 1, 2 and S1.

[b]Anaerobic growth with binary substrate mixtures (B) or crude oil (O); the respective metabolites were either detected (+, black) or not detected (-, white); a greyish filling indicates, that the compound was not tested as a co-substrate in a binary mixture experiment.

[c]Succinates were analysed as dimethyl esters via GC-MS (for eiMS data see Table S10); (I) and (II) assign chromatographically separated diastereoisomers

6. Derivatisation of diacids with (*R*)-1-phenylethanamine for stereochemical assignment

Table S4. Product formation and separability of formed diastereoisomers on two different GC columns after derivatisation with (*R*)-1-phenylethanamine.

		**Column[a]** Peaks[b]					
		**BPX-5**			**InnoWax**		
**Diacid (Stereoisomers)[c]**	**Cyclic Imide[d]**	1	2	3	1	2	3
Malonic acid (**0**)	no product	na	na	na	na	na	na
Methylsuccinic acid (**2**)	3-Methyl-1-(1-phenylethyl)pyr.	I	na	na	I	na	na
2,3-Dimethylsuccinic acid (**3**)	3,4-Dimethyl-1-(1-phenylethyl)pyr.	B	I	na	I	B	na
n-Propylsuccinic acid (**2**)	1-(1-Phenylethyl)-3-propylpyr.	B	na	na	B	na	na
Isopropylsuccinic acid (**2**)	3-Isopropyl-1-(1-phenylethyl)pyr.	B	na	na	B	na	na
n-Butylsuccinic acid (**2**)	3-Butyl-1-(1-phenylethyl)pyr.	B	na	na	B	na	na
Isobutylsuccinic acid (**2**)	3-Isobutyl-1-(1-phenylethyl)pyr.	B	na	na	B	na	na
(1-Methylpentyl)succinic acid (**4**)	3-(1-Methylpentyl)-1-(1-phenylethyl)pyr.	B	B	B	B	B	B
Cyclopentylsuccinic acid (**2**)	3-Cyclopentyl-1-(1-phenylethyl)pyr.	B	na	na	B	na	na
Cyclohexylsuccinic acid (**2**)	3-Cyclohexyl-1-(1-phenylethyl)pyr.	B	na	na	B	na	na
(Cyclohexylmethyl)succinic acid (**2**)	3-(Cyclohexylmethyl)-1-(1-phenylethyl)pyr.	B	na	na	B	na	na
Phenylsuccinic acid (**2**)	3-Phenyl-1-(1-phenylethyl)pyr.	B	na	na	N	na	na
2,3-Diphenylsuccinic acid (**3**)	3,4-Diphenyl-1-(1-phenylethyl)pyr.	B	N	na	nt	nt	na
2-Methyl-3-phenylsuccinic acid (**4**)	3-Methyl-4-phenyl-1-(1-phenylethyl)pyr.	B	B	B	nt	nt	nt
2-Phenyl-2-propylsuccinic acid (**2**)	3-Phenyl-1-(1-phenylethyl)-3-propylpyr.	B	na	na	nt	na	na
Benzylsuccinic acid (**2**)	3-Benzyl-1-(1-phenylethyl)pyr.	B	na	na	B	na	na
(1-Phenylethyl)succinic acid (**4**)	1,3-Bis-(1-phenylethyl)pyr.	B	B	B	B	B	B
(1-Phenylbutyl)succinic acid (**4**)	3-(1-Phenylbutyl)-1-(1-phenylethyl)pyr.	B	B	B	B	B	B
(1-Methyl-4-phenylbutyl)succinic acid (**4**)	3-(1-Methyl-4-phenylbutyl)-1-(1-phenylethyl)pyr.	B	B	I	nt	nt	nt
(4-Isopropylbenzyl)succinic acid (**2**)	3-(4-Isopropylbenzyl)-1-(1-phenylethyl)pyr.	B	na	na	nt	na	na
(2-Naphthylmethyl)succinic acid (**2**)	3-(2-Naphthylmethyl)-1-(1-phenylethyl)pyr.	N	na	na	N	na	na
cis-1,2-Cyclopentandicarboxylic acid (**1**)	3-(1-Phenylethyl)-3-azabicyclo[3.3.0]octane-2,4-dione	na	na	na	na	na	na
Malic acid (**2**)	3-Hydroxy-1-(1-phenylethyl)pyr.	N	na	na	N	na	na
Isopropylmalic acid (**2**)	no product	na	na	na	na	na	na
Tartaric acid (**3**)	no product	na	na	na	na	na	na
Asparagic acid (**2**)	no product	na	na	na	na	na	na
Citric acid (**0**)	no product	na	na	na	na	na	na
Isocitric acid (**4**)	no product	na	na	na	na	na	na
Glutaric acid (**0**)	no product	na	na	na	na	na	na
2-Methylglutaric acid (**2**)	no product	na	na	na	na	na	na
3-Methylglutaric acid (**0**)	no product	na	na	na	na	na	na
Camphoric acid (**3**)	no product	na	na	na	na	na	na
Glutamic acid (**2**)	no product	na	na	na	na	na	na

Baseline separation | Incomplete separation | No separation

[a]Analytical conditions were: 1. BPX-5 (SGE; 50 m, 0.22 mm i. d., 0.25 µm film thickness), 1 mL/min He, Oven: 50°C (1 min hold), 310°C (3°C/min, 30 min hold) ; 2. HPInnoWax (HP; 25 m, 0.25 mm i. d., 0.25 µm film thickness), 1.5 mL/min He, Oven; 38°C (1 min hold), 200°C (10°C/min), 250°C (8°C/min).

[b]Separation efficiency is indicated as baseline separation (B, black), incomplete separation (I, grey) or no separation (N, white) between the first and the second (1), the second and the third (2) or the third and the fourth (3) of the expected peaks of the formed diastereoisomeres.

[c]The standards were purchased from common providers or synthesized (see Materials and Methodes in the main paper); the number in brackets indicates the number of stereoisomers in the standard *viz.* the number of expected peaks.

[d]The derivatisation products were identified by GC-MS (for eiMS data see Table S10); pyr., pyrrolidine-2,5-dione.
na, not applicable; nt, not tested

7. Stereochemical assignment of succinimides formed from succinates extracted from cultures grown with crude oil

Table S5. Ratios of succinate stereoisomers detected as their respective succinimides after derivatisation with (*R*)-1-phenylethanamine in extracts of cultures grown with crude oil.

Strain[a] / Electron acceptor / **Stereoisomer ratio[b]**

Succinate[c]	Precursor	Succinimide[d]	OcN1		TD3		K172		EbN1		ToN1		mXyN1		T		Tol2		mXyS1	
			NO_3^-		SO_4^{2-}		NO_3^-		NO_3^-		NO_3^-		NO_3^-		NO_3^-		SO_4^{2-}		SO_4^{2-}	
			F	L	F	L	F	L	F	L	F	L	F	L	F	L	F	L	F	L
Isopropylsuccinate	Propane	3-Isopropyl-1-(1-phenylethyl)pyr.	0.7	1	1	1														
n-Propylsuccinate	Propane	1-(1-Phenylethyl)-3-propylpyr.			0.5	1														
(1-Methylpropyl)succinate	*n*-Butane	3-(1-Methylpropyl)-1-(1-phenylethyl)pyr.	0.5	1	1	1														
(1-Methylbutyl)succinate	*n*-Pentane	3-(1-Methylbutyl)-1-(1-phenylethyl)pyr.	0.6	1	0.6	1														
(1-Methylpentyl)succinate	*n*-Hexane	3-(1-Methylpentyl)-1-(1-phenylethyl)pyr.	0.6	1	0.4	1														
(1-Methylhexyl)succinate	*n*-Heptane	3-(1-Methylhexyl)-1-(1-phenylethyl)pyr.	0.6	1	0.5	1														
(1-Methylheptyl)succinate	*n*-Octane	3-(1-Methylheptyl)-1-(1-phenylethyl)pyr.	0.6	1	0.5	1														
(1-Methyloctyl)succinate	*n*-Nonane	3-(1-Methyloctyl)-1-(1-phenylethyl)pyr.	0.4	1	1	1														
(1-Methylnonyl)succinate	*n*-Decane	3-(1-Methylnonyl)-1-(1-phenylethyl)pyr.	0.4	1	0.6	1														
(1-Methyldecyl)succinate	*n*-Undecane	3-(1-Methyldecyl)-1-(1-phenylethyl)pyr.	0.7	1	1	1														
Cyclopentylsuccinate	Cyclopentane	3-Cyclopentyl-1-(1-phenylethyl)pyr.	1	1	0.8	1														
(Methylcyclopentyl)succinate	Methylcyclopentane	3-(Methylcyclopentyl)-1-(1-phenylethyl)pyr.	0.7	1	0.7	1														
Benzylsuccinate	Toluene	3-Benzyl-1-(1-phenylethyl)pyr.	1	1	1	1	0	1	0	1	0	1	0	1	0	1	0	1	0	1
(1-Phenylethyl)succinate	Ethylbenzene	1,3-Bis-(1-phenylethyl)pyr.	0.5	1	0.4	1														
(2-Methylbenzyl)succinate	*o*-Xylene	3-(2-Methylbenzyl)-1-(1-phenylethyl)pyr.					0	1	0	1	0	1	0	1	0	1			0	1
(3-Methylbenzyl)succinate	*m*-Xylene	3-(3-Methylbenzyl)-1-(1-phenylethyl)pyr.													0	1			0	1
(4-Methylbenzyl)succinate	*p*-Xylene	3-(4-Methylbenzyl)-1-(1-phenylethyl)pyr.	0.5	1	1	1														
(4-Ethylbenzyl)succinate	*p*-Ethyltoluene	3-(4-Ethylbenzyl)-1-(1-phenylethyl)pyr.	0.6	1	0.8	1														
[1-(4-methylphenyl)ethyl]succinate	*p*-Ethyltoluene	3-[1-(4-Methylphenyl)ethyl]-1-(1-phenylethyl)pyr.	0.2	1	1	1														
(4-Propylbenzyl)succinate	*p*-Propyltoluene	1-(1-Phenylethyl)-3-(4-propylbenzyl)pyr.	0.7	1	1	1														
(4-Isopropylbenzyl)succinate	*p*-Isopropyltoluene	3-(4-Isopropylbenzyl)-1-(1-phenylethyl)pyr.	1	1	1	1														
[1-(4-Ethylphenyl)ethyl]succinate	*p*-Diethylbenzene	3-[1-(4-Ethylphenyl)ethyl]-1-(1-phenylethyl)pyr.	0.1	1	1	1														
[1-(4-Propylphenyl)ethyl]succinate	*p*-Ethylpropylbenzene	1-(1-phenylethyl)-3-[1-(4-propylphenyl)ethyl]pyr.			1	1														
[1-(4-Isopropylphenyl)ethyl]succinate	*p*-Ethylisopropylbenzene	3-[1-(4-Isopropylphenyl)ethyl]-1-(1-phenylethyl)pyr.			1	1														

One stereoisomer | Not determined | Two stereoisomers

[a]For detailed information on the strains see Tables 1, 2 and S1.

[b]Stereoisomer ratios were determined between the first (F) and last (L) eluting peaks of the respective derivatisation products by integrating either the TIC or the M^+ trace in the chromatogram; the more intense peak was set = 1; black color indicates, that only one stereoisomer was found, white color indicates the presence of two stereoisomers.

[c]For Succinates listed in Table S2, which are missing here, stereoisomer ratios could not be determined due to the low concentrations of the analytes in the extracts (*cf.* Table S3).

[d]The derivatisation products were identified by GC-MS (for eiMS data see Table S10), pyr., pyrrolidine-2,5-dione.

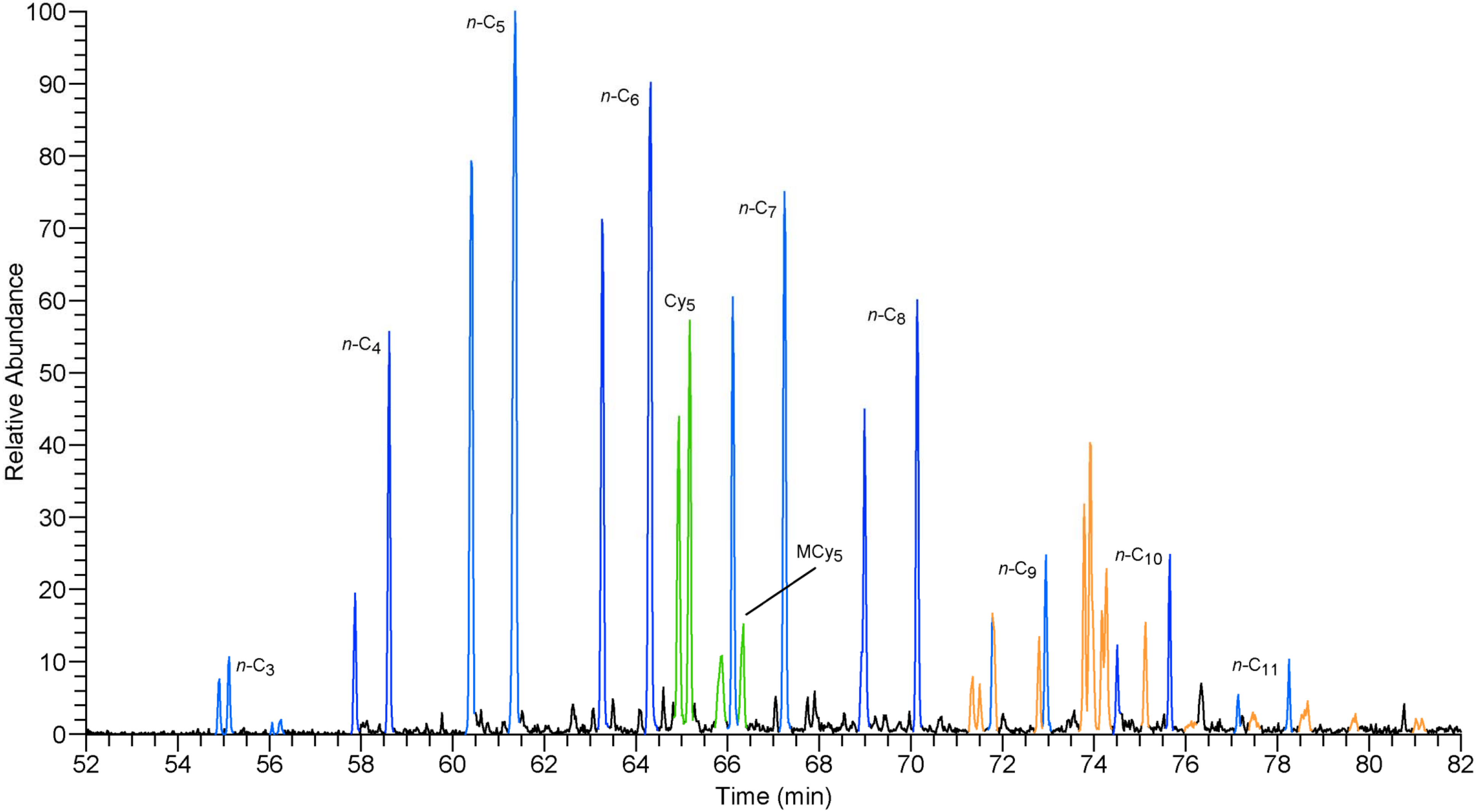

Figure S3: Partial ion chromatograms (*m/z* 202+203) of an extract of strain TD3 after anaerobic growth with crude oil. It is derivatized with (*R*)-1-phenylethanamine for separation of the stereoisomers of the alkyl-/arylalkyl-succinates. Annotated peaks represent alkylsuccinates formed from respective hydrocarbon precursors. Homologous series of activation products of *n*-alkanes in blue, cycloalkanes in green and alkylbenzenes in orange. (Annotation code: *n*-C_x: *n*-alkanes, Cy_5: cyclopentane, MCy_5: methylcyclopentane).

8. *n*-Alkylsuccinates formed by strain TD3 during growth with *n*-alkanes

Table S6. Normalized peak areas[a] of *n*-alkylsuccinates in culture extracts from strain TD3 upon growth with single *n*-alkanes.

		Growth substrate[b]					
**Succinate (as dimethyl ester)**	**Integrated mass fragment trace[c]**	***n*-Heptane**	***n*-Octane**	***n*-Nonane**	***n*-Decane**	***n*-Undecane**	***n*-Dodecane**
Methylsuccinate	59+69+87+100+101+128+129	179	248	268	426	384	551
Ethylsuccinate	55+59+69+73+83+101+114+115+142+143+146	1000	35	914	*below LOD*	1000	62
n-Propylsuccinate	55+59+69+87+97+114+115+128+129+146+157	107	1000	155	1000	164	1000
n-Butylsuccinate	87+110+114+129+138+146+171	55	11	1000	*below LOD*	660	*below LOD*
n-Pentylsuccinate	55+87+114+124+125+143+146+152+185	27	44	47	228	42	180

1000	999-500	499-200	199-100	< 100	< LOD

[a]Peak area normalized to the most intense of the peaks set = 1000 (promille); the color code represents the relative intensity of the peaks as indicated beneath the table; LOD, limit of determination.
[b]The given *n*-alkane was the sole source of carbon and energy during cultivation; for detailed information on this strain see Tables 1 and S1.
[c]The integrals from the summed fragment traces cover more than 90% of the total integral of each compound.

9. Metabolites of further transformation of selected succinates

Table S7. Examples of potential downstream metabolites derived from the respective succinates detected in culture extracts of *n* -alkane utilizers.

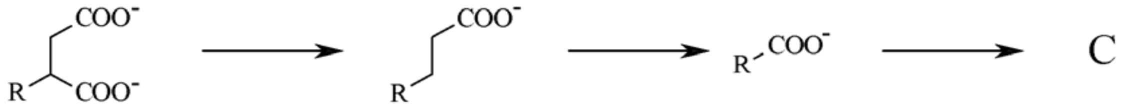

S A B

Succinate (S)	Key metabolite A[c]	Key metabolite B[d]	Further metabolite C[e]	HxN1 (NO_3^-) S	A	B	C	OcN1 (NO_3^-) S	A	B	C	TD3 (SO_4^{2-}) S	A	B	C
(1-Methylpentyl)succinate	4-Methyloctanoate	2-Methylhexanoate		+	+	+		+	+	+		+	+	+	
(1-Methylheptyl)succinate	4-Methyldecanoate	2-Methyloctanoate	Hexanoate	+	+	+	-	+	+	+	+	+	+	+	+
(1,3-Dimethylbutyl)succinate	4,6-Dimethylheptanoate	2,4-Dimethylpentanoate		+	+	+		+	+	+		+	-	+	
Cyclopentylsuccinate	3-Cyclopentylpropionate	Cyclopentylcarboxylate		+	+	-		+	-	-		+	-	-	
Benzylsuccinate	4-Phenylbutanoate	2-Phenylacetate	Benzoate	+	+	+	+	+	-	+	+	+	+	+	+
(1-Phenylethyl)succinate	4-Phenylpentanoate	2-Phenylpropionate		+	+	+		+	-	+		+	-	-	
(1-Methyl-5-phenylpentyl)succinate	4-Methyl-8-phenyloctanoate	2-Methyl-6-phenylhexanoate	4-Phenylbutanoate	+	+	-	+	+	+	+	+	+	-	-	+
(4-Methylbenzyl)succinate	4-(4-Methylphenyl)butanoate	(4-Methylphenyl)acetate	4-Methylbenzoate	+	+	+	+	+	+	+	+	+	+	+	+
[1-(4-Methylphenyl)ethyl]succinate	4-(4-Methylphenyl)pentanoate	2-(4-Methylphenyl)propionate	4-Methylbenzoate					+	-	-	+	+	-	-	-
(4-Ethylbenzyl)succinate	4-(4-Ethylphenyl)butanoate	(4-Ethylphenyl)acetate	4-Ethylbenzoate					+	+	+	+	+	+	+	+

Column header: Strain[a] / Electron acceptor / Metabolite[b]

Legend: Detected (+) | Not tested | Not detected (-)

[a]For detailed information on the strains and their specific main substrates see Tables 1, 2 and S1.

[b]Metabolites were identified as methyl esters by standard comparison, by comparison with libary spectra or based on their chromatographic and mass spectrometric behavior (see Table S10); they were detected (+, black) or not (-, white) in the respective extracts.

[c]Respective metabolite formed upon activation as coenzyme A ester, intramolecular rearrangement and subsequent decarboxylation.

[d]Respective metabolite formed upon first round of β-oxidation.

[e]Further metabolite depending on the actual substrate and transformation pathway (Rabus et al. 2001 & 2010; Wilkes et al. 2002, 2003).

Table S8. Examples of potential downstream metabolites derived from the respective succinates detected in culture extracts of alkylbenzene utilizers.

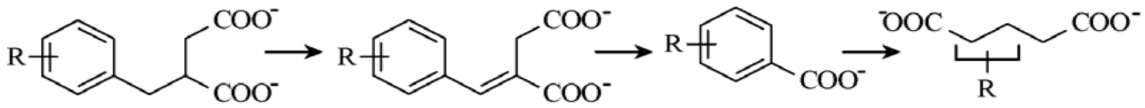

S I B G

Succinate (S)	Itaconate (I)	Benzoate (B)	Glutarate (G)
Benzylsuccinate	Phenylitaconate	Benzoate	Glutarate
(2-Methylbenzylsuccinate)	(2-Methylphenyl)itaconate	2-Methylbenzoate	
(3-Methylbenzylsuccinate)	(3-Methylphenyl)itaconate	3-Methylbenzoate	2-Methylglutarate
(4-Methylbenzylsuccinate)	(4-Methylphenyl)itaconate	4-Methylbenzoate	3-Methylglutarate
(3-Ethylbenzyl)succinate	(3-Ethylphenyl)itaconate	3-Ethylbenzoate	2-Ethylglutarate
(3,5-Dimethylbenzyl)succinate	(3,5-Dimethylphenyl)itaconate	3,5-Dimethylbenzoate	2,4-Dimethylglutarate

Strain[a] / Electron acceptor / Metabolite[b]

Succinate (S)	K172 (NO_3^-) S	I	B	G	EbN1 (NO_3^-) S	I	B	G	ToN1 (NO_3^-) S	I	B	G	mXyN1 (NO_3^-) S	I	B	G
Benzylsuccinate	+	+	+	+	+	+	+	+	+	+	+	+	+	+	+	+
(2-Methylbenzylsuccinate)					+	+	+						+	+	+	
(3-Methylbenzylsuccinate)					+	+	+	+					+	+	+	+
(4-Methylbenzylsuccinate)					-	-	+	-					+	+	+	+
(3-Ethylbenzyl)succinate					-	-	+	-								
(3,5-Dimethylbenzyl)succinate																

Succinate (S)	T (NO_3^-) S	I	B	G	Tol2 (SO_4^{2-}) S	I	B	G	oXyS1 (SO_4^{2-}) S	I	B	G	mXyS1 (SO_4^{2-}) S	I	B	G
Benzylsuccinate	+	+	+	+	+	+	+	+	+	+	+	+	+	+	+	-
(2-Methylbenzylsuccinate)	+	+	+		+	+	+		+	+	+		+	-	+	
(3-Methylbenzylsuccinate)	+	+	+	+	+	-	+	+	-	-	+	+	+	+	+	+
(4-Methylbenzylsuccinate)	+	+	+	-	+	+	+	-					+	+	+	+
(3-Ethylbenzyl)succinate													+	+	+	+
(3,5-Dimethylbenzyl)succinate													+	+	+	+

Detected (+) | Not tested | Not detected (-)

[a]For detailed information on the strains and their specific main substrates see Tables 1, 2 and S1.

[b]Metabolites were identified as methyl esters by standard comparison, by comparison with libary spectra or based on their chromatographic and mass spectrometric behavior (see Table S10); they were detected (black, +) or not (white, -) in the respective extracts.

10. Further activation products of hydrocarbons

Table S9. Further activation products of hydrocarbons identified in culture extracts upon growth with binary mixtures.

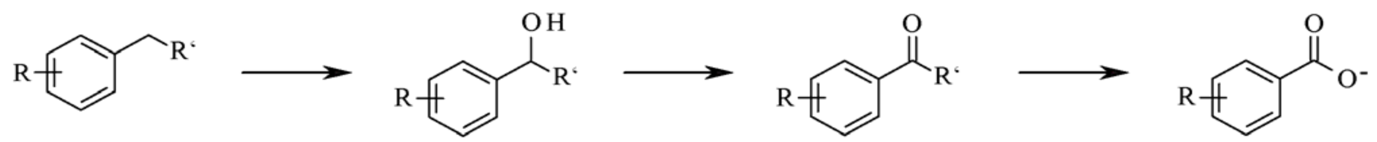

				Strain[a] / Electron acceptor / Activation product[b]																																
				HxN1			OcN1			TD3			K172			EbN1			ToN1			mXyN1			T			Tol2			oXyS1			mXyS1		
				NO_3^-			NO_3^-			SO_4^{2-}			NO_3^-			NO_3^-			NO_3^-			NO_3^-			NO_3^-			SO_4^{2-}			SO_4^{2-}			SO_4^{2-}		
**Precursor (Position)**	**Alcohol (H)**	**Oxo-compound (O)**	**Acid (A)**	**H**	**O**	**A**	**H**	**O**	**A**	**H**	**O**	**A**	**H**	**O**	**A**	**H**	**O**	**A**	**H**	**O**	**A**	**H**	**O**	**A**	**H**	**O**	**A**	**H**	**O**	**A**	**H**	**O**	**A**	**H**	**O**	**A**
Toluene	Benzyl alcohol	Benzaldehyde	Benzoate	+	-	+	+	+	+	+	+	+	+	+	+	+	-	+	+	+	+	-	-	+	-	-	+	-	+	+	-	+	+	-	-	+
Ethylbenzene (benzyl)	1-Phenylethan-1-ol	Acetophenone	Benzoate	+	+	+	+	+	+	-	+	+				+	+	+																		
Propylbenzene (benzyl)	1-Phenylpropan-1-ol	Propiophenone	Benzoate	+	+	+	+	+	+	+	+	+																								
Butylbenzene (benzyl)	1-Phenylbutan-1-ol	Butyrophenone	Benzoate	+	+	+	+	+	+	-	-	+							-	-	+							-	-	+						
Butylbenzene (2')	1-Phenylbutan-2-ol	Benzyl ethyl ketone	Phenylacetate	-	+	+	-	+	+	-	-	+							-	-	-							-	-	-						
Pentylbenzene (benzyl)	1-Phenylpentanol	Butyl phenyl ketone	Benzoate	-	-	+	-	-	+	-	-	+							-	-	+							-	-	+						
o-Xylene	2-Methylbenzyl alcohol	2-Methylbenzaldehyde	2-Methylbenzoate	-	-	+	+	+	-	-	+	+				+	+	+				+	-	+	-	-	+	-	-	+	-	-	+	-	-	+
o-Ethyltoluene (methyl)	2-Ethylbenzyl alcohol	2-Ethylbenzaldehyde	2-Ethylbenzoate				+	+	+	+	+	+				+	+	+													-	+	+			
o-Ethyltoluene (1'-ethyl)	(2-Methylphenyl)ethan-1-ol	2-Methylacetophenone	2-Methylbenzoate				+	+	+	+	+	+				+	+	+													-	+	+			
o-Isopropyltoluene (methyl)	2-Isopropylbenzyl alcohol	2-Isopropylbenzaldehyde	2-Isopropylbenzoate				-	-	+	+	-	+																								
m-Xylene	3-Methylbenzyl alcohol	3-Methylbenzaldehyde	3-Methylbenzoate	-	-	+	-	-	+	-	-	+				-	-	+				-	-	+	-	-	+	-	-	+	-	-	+	-	-	+
m-Ethyltoluene (methyl)	3-Ethylbenzyl alcohol	3-Ethylbenzaldehyde	3-Ethylbenzoate				-	-	+	-	-	+				-	-	+																-	-	+
m-Ethyltoluene (1'-ethyl)	(3-Methylphenyl)ethan-1-ol	3-Methylacetophenone	3-Methylbenzoate				+	+	+	+	+	+				+	+	+																-	-	+
m-Isopropyltoluene (methyl)	3-Isopropylbenzyl alcohol	3-Isopropylbenzaldehyde	3-Isopropylbenzoate				-	-	+	+	-	+																								
p-Xylene	4-Methylbenzyl alcohol	4-Methylbenzaldehyde	4-Methylbenzoate	-	+	+	-	+	+	-	+	+				-	+	+				-	+	+	-	-	+	-	-	+				-	+	+
p-Ethyltoluene (methyl)	4-Ethylbenzyl alcohol	4-Ethylbenzaldehyde	4-Ethylbenzoate				-	+	+	-	-	+				-	-	+																		
p-Ethyltoluene (1'-ethyl)	4-Methylphenylethan-1-ol	4-Methylacetophenone	4-Methylbenzoate				+	+	+	-	+	-				+	+	+																		
p-Isopropyltoluene (methyl)	4-Isopropylbenzyl alcohol	4-Isopropylbenzaldehyde	4-Isopropylbenzoate				-	-	+	-	-	+																								
p-*tert*-Butyltoluene (methyl)	4-*tert*-Butylbenzyl alcohol	4-*tert*-Butylbenzaldehyde	4-*tert*-Butylbenzoate				-	-	+	-	-	+																								
1,2,3-Trimethylbenzene (1')	2,3-Dimethylbenzyl alcohol	2,3-Dimethylbenzaldehyde	2,3-Dimethylbenzoate				+	+	+	+	+	+																								
1,2,3-Trimethylbenzene (2')	2,6-Dimethylbenzyl alcohol	2,6-Dimethylbenzaldehyde	2,6-Dimethylbenzoate				+	+	+	+	+	+																								
1,2,4-Trimethylbenzene (1')	2,4-Dimethylbenzyl alcohol	2,4-Dimethylbenzaldehyde	2,4-Dimethylbenzoate				+	+	-	+	+	+																			+	+	+	+	+	+
1,2,4-Trimethylbenzene (2')	2,5-Dimethylbenzyl alcohol	2,5-Dimethylbenzaldehyde	2,5-Dimethylbenzoate				+	+	-	+	+	+																			+	+	+	+	+	+
1,2,4-Trimethylbenzene (4')	3,4-Dimethylbenzyl alcohol	3,4-Dimethylbenzaldehyde	3,4-Dimethylbenzoate				+	+	+	+	+	+																			+	+	+	+	+	+
1,3,5-Trimethylbenzene (1')	3,5-Dimethylbenzyl alcohol	3,5-Dimethylbenzaldehyde	3,5-Dimethylbenzoate				-	-	-	-	-	-																						-	-	+
1,2,3,5-Tetramethylbenzene (1')	2,3,5-Trimethylbenzyl alcohol	2,3,5-Trimethylbenzaldehyde	2,3,5-Trimethylbenzoate				+	+	+	+	+	+																								
1,2,3,5-Tetramethylbenzene (2')	2,4,6-Trimethylbenzyl alcohol	2,4,6-Trimethylbenzaldehyde	2,4,6-Trimethylbenzoate				+	+	+	+	+	+																								
1,2,3,5-Tetramethylbenzene (5')	3,4,5-Trimethylbenzyl alcohol	3,4,5-Trimethylbenzaldehyde	3,4,5-Trimethylbenzoate				+	+	+	+	+	+																								
1,2,4,5-Tetramethylbenzene (1')	2,4,5-Trimethylbenzyl alcohol	2,4,5-Trimethylbenzaldehyde	2,4,5-Trimethylbenzoate				+	+	+	+	+	+																								
Pentamethylbenzene (1')	2,3,4,5-Tetramethylbenzyl alcohol	2,3,4,5-Tetramethylbenzaldehyde	2,3,4,5-Tetramethylbenzoate				+	+	+	+	-	+																								
Pentamethylbenzene (2')	2,3,4,6-Tetramethylbenzyl alcohol	2,3,4,6-Tetramethylbenzaldehyde	2,3,4,6-Tetramethylbenzoate				+	+	+	+	+	+																								
Pentamethylbenzene (3')	2,3,5,6-Tetramethylbenzyl alcohol	2,3,5,6-Tetramethylbenzaldehyde	2,3,5,6-Tetramethylbenzoate				+	+	+	+	-	+																								
Hexamethylbenzene	Pentamethylbenzylalcohol	Pentamethylbenzaldehyde	Pentamethylbenzoate				+	+	+	+	+	+																								

Detected (+) | Not tested | Not detected (-)

[a]For detailed information on the strains and their specific main substrates see Tables 1, 2 and S1.

[b]Metabolites were identified (acids as methyl esters) by standard comparison, by comparison with libary spectra or based on their chromatographic and mass spectrometric behavior (see Table S10); they were detected (+, black) or not (-, white) in the respective extracts; the lack of an adequate culture is indicated by a greyish filling.

11. eiMS data of identified metabolites

Table S10. Identified compounds (acids as methyl esters) and their eiMS key ions.

	MW	Key ions (eiMS)
**Alcohols**		
Benzyl alcohol*	108	108, 107, 79, 77, 51
1-Phenylethan-1-ol	122	122, 107, 104, 79, 77, 51
1-Phenylpropan-1-ol	136	136, 117, 107, 79, 77, 51
1-Phenylbutan-1-ol	150	150, 117, 107, 79, 77
1-Phenylpentanol	164	164, 107, 79, 77
2-Methylbenzyl alcohol	122	122, 107, 104, 91, 79, 77
2-Ethylbenzyl alcohol	136	136, 118, 117, 107, 91, 79, 77
(2-Methylphenyl)ethan-1-ol	136	136, 121, 118, 117, 93, 91, 77
2-Isopropylbenzyl alcohol	150	150, 135, 119, 107, 105, 91, 79, 77
3-Methylbenzyl alcohol	122	122, 107, 104, 91, 79, 77
3-Ethylbenzyl alcohol	136	136, 107, 91, 79, 77
(3-Methylphenyl)ethan-1-ol	136	136, 121, 93, 91, 77
3-Isopropylbenzyl alcohol	150	150, 135, 119, 107, 105, 91, 79, 77
4-Methylbenzyl alcohol	122	122, 107, 104, 91, 79, 77
4-Ethylbenzyl alcohol	136	136, 107, 91, 79, 77
4-Methylphenylethan-1-ol	136	136, 121, 93, 91, 77
4-Isopropylbenzyl alcohol	150	150, 135, 119, 107, 105, 91, 79, 77
2,3-Dimethylbenzylalcohol	136	136, 121, 118, 117, 107, 105, 93, 91, 77
2,6-Dimethylbenzylalcohol	136	136, 121, 118, 117, 107, 105, 93, 91, 77
2,4-Dimethylbenzylalcohol	136	136, 121, 118, 107, 105, 93, 91, 77
2,5-Dimethylbenzylalcohol	136	136, 121, 118, 107, 105, 93, 91, 77
3,4-Dimethylbenzylalcohol	136	136, 121, 107, 105, 93, 91, 77
3,5-Dimethylbenzyl alcohol	136	136, 121, 107, 105, 93, 91, 77
2,3,5-Trimethylbenzylalcohol	150	150, 132, 107, 91, 77
2,4,6-Trimethylbenzylalcohol	150	150, 132, 107, 91, 77
3,4,5-Trimethylbenzylalcohol	150	150, 132, 107, 91, 77
2,4,5-Trimethylbenzylalcohol	150	150, 132, 107, 91, 77
2,3,4,5-Tetramethylbenzylalcohol	164	164, 146, 131, 121, 105, 91
2,3,4,6-Tetramethylbenzylalcohol	164	164, 146, 131, 121, 105, 91
2,3,5,6-Tetramethylbenzylalcohol	164	164, 146, 131, 121, 105, 91
Pentamethylbenzylalcohol	178	178, 160, 145
**Oxo-compounds**		
Benzaldehyde*	106	106, 105, 77, 51
Acetophenone*	120	120, 105, 77, 51
Propiophenone	134	134, 105, 77, 51
Butyrophenone	148	148, 120, 105, 77, 51
Benzyl ethyl ketone	148	148, 91, 65, 57
Butyl phenyl ketone	162	162, 120, 105, 77, 51
2-Methylbenzaldehyde	120	120, 119, 91
2-Ethylbenzaldehyde	134	134, 133, 105, 91
2-Methylacetophenone	134	134, 119, 91, 65
2-Isopropylbenzaldehyde	148	148, 133, 119, 105, 91, 77
3-Methylbenzaldehyde	120	120, 119, 91
3-Ethylbenzaldehyde	134	134, 133, 119, 105, 91, 77
3-Methylacetophenone	134	134, 119, 91, 65
3-Isopropylbenzaldehyde	148	148, 133, 119, 105, 91, 77
4-Methylbenzaldehyde	120	120, 119, 91
4-Ethylbenzaldehyde	134	134, 133, 119, 105, 91, 77
4-Methylacetophenone	134	134, 119, 91, 65
4-Isopropylbenzaldehyde	148	148, 133, 119, 105, 91, 77
2,3-Dimethylbenzaldehyd	134	134, 133, 115, 103, 91, 79, 77
2,6-Dimethylbenzaldehyd	134	134, 133, 115, 103, 91, 79, 77
2,4-Dimethylbenzaldehyd	134	134, 133, 105
2,5-Dimethylbenzaldehyd	134	134, 133, 105
3,4-Dimethylbenzaldehyd	134	134, 133, 105
3,5-Dimethylbenzaldehyde	134	134, 133, 105
2,3,5-Trimethylbenzaldehyde	148	148, 147, 119, 105, 91

Table S10. Continued.

	MW	Key ions (eiMS)
**Oxo-compounds (continued)**		
2,4,6-Trimethylbenzaldehyde	148	148, 147, 119, 105, 91
3,4,5-Trimethylbenzaldehyde	148	148, 147, 119, 105, 91
2,4,5-Trimethylbenzaldehyd	148	148, 147, 119, 105, 91
2,3,4,5-Tetramethylbenzaldehyd	162	162, 161, 133, 119, 105, 91
2,3,4,6-Tetramethylbenzaldehyd	162	162, 161, 133, 119, 105, 91
2,3,5,6-Tetramethylbenzaldehyd	162	162, 161, 133, 119, 105, 91
Pentamethylbenzaldehyd	176	176, 175, 147, 133
**Monoacids (as methyl esters)**		
Benzoate*	136	136, 105, 77, 51
Phenylacetate*	150	150, 91
2-Methylbenzoate*	150	150, 119, 118, 91, 90
2-Ethylbenzoate*	164	164, 149, 133, 132, 131, 105, 104, 103, 91, 79, 77, 51
2-Isopropylbenzoate	178	178, 163, 147, 131, 119, 103, 91, 77, 59
3-Methylbenzoate*	150	150, 119, 118, 91, 90
3-Ethylbenzoate	164	164, 149, 133, 105, 79, 77
3-Isopropylbenzoate	178	178, 163, 147, 131, 119, 103, 91, 77, 59
4-Methylbenzoate*	150	150, 119, 118, 91, 90
4-Ethylbenzoate*	164	164, 149, 133, 105, 79, 77
4-Isopropylbenzoate*	178	178, 163, 147, 131, 119, 103, 91, 77, 59
4-*tert* -Butylbenzoate	192	192, 178, 177, 161, 149
2,3-Dimethylbenzoate*	164	164, 149, 133, 132, 105, 104, 77
2,6-Dimethylbenzoate*	164	164, 149, 133, 132, 105, 104, 77
2,4-Dimethylbenzoate*	164	164, 149, 133, 132, 105, 104, 103, 77
2,5-Dimethylbenzoate*	164	164, 149, 133, 132, 105, 104, 103, 77
3,4-Dimethylbenzoate*	164	164, 133, 105, 77
3,5-Dimethylbenzoate*	164	164, 133, 105, 77
2,3,5-Trimethylbenzoate	178	178, 147, 146, 119, 118, 91
2,4,6-Trimethylbenzoate	178	178, 147, 146, 119, 118, 91
3,4,5-Trimethylbenzoate	178	178, 147, 119, 118, 91
2,4,5-Trimethylbenzoate	178	178, 147, 146, 119, 118, 91
2,3,4,5-Tetramethylbenzoate	192	192, 177, 161, 160, 133, 117, 115, 105, 91, 77
2,3,4,6-Tetramethylbenzoate	192	192, 177, 161, 160, 133, 117, 115, 105, 91, 77
2,3,5,6-Tetramethylbenzoate	192	192, 177, 161, 160, 133, 117, 115, 105, 91, 77
Pentamethylbenzoate	206	206, 191, 175, 174, 147, 146, 131
4-Methyloctanoate*	172	143, 141, 115, 99, 87, 74
4-Methyldecanoate	200	171, 169, 143, 127, 115, 87, 74
4,6-Dimethylheptanoate	172	143, 141, 123, 115, 99, 87, 74, 69
3-Cyclopentylpropionate*	156	156, 141, 125, 87, 83, 74, 69, 67, 55
4-Phenylbutanoate*	178	178, 147, 146, 105, 104, 91, 74, 65
4-Phenylpentanoate	192	192, 161, 160, 119, 118, 105, 91, 79, 77, 74
4-Methyl-8-phenyloctanoate	248	248, 217, 216, 199, 175, 119, 117, 105, 104, 91, 87, 74, 65, 55
4-(4-Methylphenyl)butanoate	192	192, 161, 118, 105, 74
4-(4-Ethylphenyl)butanoate	206	206, 175, 147, 133, 132, 119, 117, 104, 91, 74
2-Methylhexanoate*	144	144, 129, 113, 101, 88
2-Methyloctanoate	172	172, 157, 141, 129, 115, 101, 88
2,4-Dimethylpentanoate	144	129, 113, 101, 88, 73, 69, 59
2-Phenylpropionate*	164	164, 105, 79, 77
2-Methyl-6-phenylhexanoate	220	220, 188, 130, 104, 101, 91, 88
(4-Methylphenyl)acetate*	164	164, 105, 77
(4-Ethylphenyl)acetate	178	178, 163, 119, 104, 91, 77
Hexanoate*	130	101, 99, 87, 74, 59

Table S10. Continued.

	MW	Key ions (eiMS)
**Succinates (as dimethyl esters)**		
Methylsuccinate*	160	129, 128, 101, 100, 87, 69, 59
Ethylsuccinate	174	146, 143, 114, 101, 83, 73, 59, 55
Isopropylsuccinate*	188	157, 146, 115, 114
n -Propylsuccinate*	188	157, 146, 129, 128, 115, 114
(1-Methylpropyl)succinate (I)	202	171, 146, 129, 114
(1-Methylpropyl)succinate (II)	202	171, 146, 129, 114
n -Butylsuccinate*	202	171, 146, 138, 129, 114
(1-Ethylpropyl)succinate	216	185, 146, 143, 114
(1-Methylbutyl)succinate (I)	216	185, 146, 143, 114
(1-Methylbutyl)succinate (II)	216	185, 146, 143, 114
n -Pentylsuccinate	216	185, 152, 146, 143, 125, 124, 114
(1-Ethylbutyl)succinate*	230	199, 157, 146, 114
(1-Methylpentyl)succinate* (I)	230	199, 157, 146, 114
(1-Methylpentyl)succinate* (II)	230	199, 157, 146, 114
n -Hexylsuccinate	230	199, 157, 146, 114
(1-Ethylpentyl)succinate	244	213, 171, 146, 114
(1-Methylhexyl)succinate (I)	244	213, 171, 146, 114
(1-Methylhexyl)succinate (II)	244	213, 171, 146, 114
(1-Ethylhexyl)succinate	258	227, 185, 146, 114
(1-Methylheptyl)succinate (I)	258	227, 185, 146, 114
(1-Methylheptyl)succinate (II)	258	227, 185, 146, 114
(1-Ethylheptyl)succinate	272	241, 199, 146, 114
(1-Methyloctyl)succinate (I)	272	241, 199, 146, 114
(1-Methyloctyl)succinate (II)	272	241, 199, 146, 114
(1-Ethyloctyl)succinate	286	255, 213, 146, 114
(1-Methylnonyl)succinate	286	255, 213, 146, 114
(1-Ethylnonyl)succinate	300	269, 227, 146, 114
(1-Methyldecyl)succinate	300	269, 227, 146, 114
(1-Ethyldecyl)succinate	314	283, 241, 146, 114
(1-Methylundecyl)succinate	314	283, 241, 146, 114
(1-Ethylundecyl)succinate	328	297, 255, 146, 114
(1-Methyldodecyl)succinate	328	297, 255, 146, 114
(1-Methyltridecyl)succinate	342	311, 269, 146, 114
(1-Methylpentadecyl)succinate	370	339, 297, 146, 114
(1,3-Dimethylbutyl)succinate	230	199, 157, 146, 114
(br-C_7)succinate	244	213, 171, 146, 114
likely (1,4-Dimethylpentyl)succinate (I)	244	213, 171, 146, 114
likely (1,4-Dimethylpentyl)succinate (II)	244	213, 171, 146, 114
(br-C_8)succinate 1	258	227, 185, 146, 114
(br-C_8)succinate 2	258	227, 185, 146, 114
(br-C_8)succinate 3	258	227, 185, 146, 114
(br-C_8)succinate 4	258	227, 185, 146, 114
likely (1,5-Dimethylhexyl)succinate (I)	258	227, 185, 146, 114
likely (1,5-Dimethylhexyl)succinate (II)	258	227, 185, 146, 114
(br-C_9)succinate	272	241, 199, 146, 114
Cyclopentylsuccinate*	214	183, 150, 146, 141, 123, 114
(Methylcyclopentyl)succinate	228	197, 155, 146, 136, 114
Succinate from Ethylcyclopentane 1	242	211, 169, 146, 114
Succinate from Ethylcyclopentane 2	242	211, 171, 169, 146, 114
Succinate from Ethylcyclopentane 3	242	211, 169, 146, 114
Succinate from Ethylcyclopentane 4	242	211, 171, 169, 146, 114
Cyclohexylsuccinate*	228	197, 169, 155, 146, 137, 114
Benzylsuccinate*	236	236, 176, 173, 163, 145, 131, 117, 91
(1-Phenylethyl)succinate* (I)	250	250, 190, 177, 159, 145, 131, 105
(1-Phenylethyl)succinate* (II)	250	250, 190, 177, 159, 145, 131, 105
(1-Phenylpropyl)succinate	264	264, 233, 232, 204, 201, 191, 173, 159, 145, 119, 117, 115, 91
(1-Methyl-2-phenylethyl)succinate (I)	264	264, 233, 232, 204, 201, 191, 173, 159, 145, 119, 91
(1-Methyl-2-phenylethyl)succinate (II)	264	264, 233, 232, 204, 201, 191, 173, 159, 145, 119, 91
(1-Methyl-3-phenylpropyl)succinate*	278	278, 246, 214, 187, 145, 131, 114, 104, 91
(1-Ethyl-3-phenylpropyl)succinate	292	292, 260, 228, 216, 204, 171, 147, 125, 116, 91, 69
(1-Methyl-4-phenylbutyl)succinate*	292	292, 260, 228, 201, 144, 129, 114, 104, 91, 55

Table S10. Continued.

	MW	Key ions (eiMS)
**Succinates (as dimethyl esters, continued)**		
(1-Ethyl-4-phenylbutyl)succinate	306	306, 274, 242, 215, 158, 146, 129, 114, 104, 91
(1-Methyl-5-phenylpentyl)succinate	306	306, 275, 274, 256, 242, 215, 201, 183, 182, 146, 141, 114, 104, 91
(1-Ethyl-5-phenylpentyl)succinate	320	320, 288, 270, 256, 215, 197, 196, 179, 155, 146, 114, 104, 91
(1-Methyl-6-phenylhexyl)succinate	320	320, 289, 288, 270, 256, 197, 184, 152, 146, 141, 117, 114, 104, 91
(1-Ethyl-6-phenylhexyl)succinate	334	334, 303, 302, 273, 225, 211, 198, 166, 155, 146, 114, 104, 91
(1-Methyl-7-phenylheptyl)succinate	334	334, 303, 302, 284, 270, 229, 210, 166, 146, 114, 104, 91
(1-Ethyl-7-phenylheptyl)succinate	348	348, 317, 316, 298, 287, 255, 243, 224, 155, 146, 131, 114, 104, 91
(1-Methyl-8-phenyloctyl)succinate	348	348, 316, 284, 257, 256, 243, 224, 146, 131, 114, 104, 91
(1-Ethyl-8-phenyloctyl)succinate	362	362, 330, 298, 289, 281, 269, 257, 146, 114, 104, 91
(1-Methyl-9-phenylnonyl)succinate	362	362, 331, 330, 298, 270, 257, 228, 146, 131, 114, 104, 91
(2-Methylbenzyl)succinate	250	250, 232, 219, 218, 190, 187, 177, 159, 145, 131, 117, 116, 115, 105, 91
(2-Ethylbenzyl)succinate	264	264, 246, 233, 204, 200, 191, 190, 173, 159, 145, 119, 117, 115, 104, 91
(3-Methylbenzyl)succinate	250	250, 219, 190, 187, 177, 159, 145, 131, 117, 115, 105, 91
[1-(3-Methylphenyl)ethyl]succinate (I)	264	264, 233, 204, 200, 191, 173, 159, 145, 131, 119, 91
[1-(3-Methylphenyl)ethyl]succinate (II)	264	264, 233, 204, 200, 191, 173, 159, 145, 131, 119, 91
(3-Ethylbenzyl)succinate	264	264, 233, 204, 200, 191, 190, 173, 159, 145, 119, 117, 115, 104, 91
(3-Isopropylbenzyl)succinate	278	278, 263, 247, 218, 215, 205, 203, 189, 187, 173, 159, 145, 133, 131, 117, 115, 105, 91
(3-Propylbenzyl)succinate	278	278, 247, 218, 215, 205, 187, 179, 173, 159, 133, 131, 117, 115, 91
(4-Methylbenzyl)succinate	250	250, 219, 190, 187, 177, 159, 145, 131, 117, 115, 105, 91
[1-(4-Methylphenyl)ethyl]succinate (I)	264	264, 233, 204, 200, 191, 173, 159, 145, 131, 119, 91
[1-(4-Methylphenyl)ethyl]succinate (II)	264	264, 233, 204, 200, 191, 173, 159, 145, 131, 119, 91
(4-Ethylbenzyl)succinate	264	264, 233, 204, 200, 191, 190, 173, 159, 145, 119, 117, 115, 104, 91
[1-(4-Ethylphenyl)ethyl]succinate (I)	278	278, 247, 218, 215, 205, 187, 173, 159, 145, 133, 117, 105, 91
[1-(4-Ethylphenyl)ethyl]succinate (II)	278	278, 247, 218, 215, 205, 187, 173, 159, 145, 133, 117, 105, 91
(4-Isopropylbenzyl)succinate*	278	278, 263, 247, 218, 215, 205, 203, 189, 187, 173, 159, 145, 133, 131, 117, 115, 105, 91
(4-Propylbenzyl)succinate	278	278, 247, 218, 215, 205, 189, 187, 175, 173, 159, 133, 117, 115, 104, 91
[1-(4-Isopropylphenyl)ethyl]succinate (I)	292	292, 261, 232, 219, 201, 187, 147, 131, 117, 105, 91
[1-(4-Isopropylphenyl)ethyl]succinate (II)	292	292, 261, 232, 219, 201, 187, 147, 131, 117, 105, 91
[1-(4-Propylphenyl)ethyl]succinate (I)	292	292, 261, 232, 219, 201, 192, 187, 173, 147, 131, 117, 105, 91
[1-(4-Propylphenyl)ethyl]succinate (II)	292	292, 261, 232, 219, 201, 192, 187, 173, 147, 131, 117, 105, 91
(4-*tert* -Butylbenzyl)succinate	292	292, 277, 261, 245, 232, 229, 219, 217, 213, 203, 201, 187, 185, 163, 147, 132, 131,
(3-C_4-benzyl)succinate	292	292, 261, 232, 229, 219, 201, 187, 173, 159, 155, 144, 131, 129, 117, 115, 113, 105, 91
(3-C_4-benzyl)succinate	292	292, 261, 232, 221, 219, 203, 201, 199, 189, 187, 171, 159, 145, 143, 131, 129, 117, 115, 91
(4-C_4-benzyl)succinate	292	292, 261, 232, 229, 219, 201, 189, 187, 175, 147, 131, 115, 104, 91
[1-(4-C_4-phenyl)ethyl]succinate (I)	306	306, 275, 263, 246, 233, 215, 203, 201, 187, 161, 131, 129, 117, 105, 91
[1-(4-C_4-phenyl)ethyl]succinate (II)	306	306, 275, 263, 246, 233, 215, 203, 201, 187, 161, 131, 129, 117, 105, 91
(4-Butylbenzyl)succinate	292	292, 261, 232, 229, 219, 218, 201, 189, 187, 175, 173, 147, 131, 117, 104, 91
[1-(4-Butylphenyl)ethyl]succinate (I)	306	306, 275, 263, 246, 233, 215, 203, 201, 187, 161, 131, 129, 117, 105, 91
[1-(4-Butylphenyl)ethyl]succinate (II)	306	306, 275, 263, 246, 233, 215, 203, 201, 187, 161, 131, 129, 117, 105, 91
(4-Pentylbenzyl)succinate	306	306, 275, 246, 243, 233, 215, 201, 189, 161, 131, 117, 104, 91
(2,6-Dimethylbenzyl)succinate	264	264, 246, 233, 204, 191, 173, 159, 145, 119, 117, 91
(2,3-Dimethylbenzyl)succinate	264	264, 246, 233, 204, 191, 173, 159, 145, 119, 117, 91
(2,5-Dimethylbenzyl)succinate	264	264, 246, 233, 232, 204, 201, 200, 191, 173, 159, 145, 119, 117, 91
(2,4-Dimethylbenzyl)succinate	264	264, 246, 233, 232, 204, 201, 200, 191, 173, 159, 145, 119, 117, 91
(3,4-Dimethylbenzyl)succinate	264	264, 233, 232, 204, 201, 191, 173, 159, 145, 119, 117, 91
(3,5-Dimethylbenzyl)succinate	264	264, 233, 204, 191, 173, 159, 145, 119, 117, 91
(1-Naphthylmethyl)succinate	286	286, 226, 223, 212, 205, 195, 181, 167, 165, 153, 152, 141, 128, 115, 97
(2-Naphthylmethyl)succinate*	286	286, 226, 223, 212, 205, 195, 181, 167, 165, 153, 152, 141, 128, 115, 97
**Itaconates (as dimethyl esters)**		
Phenylitaconate	234	234, 203, 202, 175, 174, 131, 115, 91, 77
(2-Methylphenyl)itaconate	248	248, 217, 216, 185, 184, 157, 156, 145, 129, 115
(3-Methylphenyl)itaconate	248	248, 217, 216, 189, 188, 157, 145, 129, 115
(4-Methylphenyl)itaconate	248	248, 217, 216, 189, 188, 157, 145, 129, 115
(3-Ethylphenyl)itaconate	262	262, 231, 230, 203, 202, 171, 159, 143, 129, 128, 115
(3,5-Dimethylphenyl)itaconate	262	162, 231, 230, 220, 203, 202, 171, 144, 143, 129, 128, 115

Table S10. Continued.

	MW	Key ions (eiMS)
**Glutarates (as dimethyl esters)**		
Glutarate*	160	129, 128, 101, 100, 87, 74, 59, 55
2-Methylglutarate*	174	143, 142, 115, 114, 101, 99, 88, 83, 73, 59, 55
3-Methylglutarate*	174	143, 142, 115, 114, 101, 82, 74, 73, 69, 59, 55
2-Ethylglutarate	188	157, 129, 128, 113, 102, 100, 97, 87, 74, 69, 59
2,4-Dimethylglutarate	188	157, 129, 128, 113, 101, 97, 88, 73, 69, 59
**Succinimides**		
3-Methyl-1-(1-phenylethyl)pyrrolidine-2,5-dione	217	217, 174, 146, 132, 120, 106, 105, 104, 103, 79, 78, 77, 69, 51
3,4-Dimethyl-1-(1-phenylethyl)pyrrolidine-2,5-dione	231	231, 188, 146, 132, 120, 105, 104, 103, 79, 77, 69, 56, 54
1-(1-Phenylethyl)-3-propylpyrrolidine-2,5-dione	245	245, 230, 203, 202, 146, 132, 120, 105, 104, 103, 99, 77, 55
3-Isopropyl-1-(1-phenylethyl)pyrrolidine-2,5-dione	245	245, 230, 203, 202, 174, 161, 160, 146, 132, 120, 104, 99, 79, 77, 55
3-Butyl-1-(1-phenylethyl)pyrrolidine-2,5-dione	259	259, 244, 216, 203, 189, 188, 174, 160, 146, 132, 120, 106, 105, 104, 103, 99, 91, 77, 55
3-Isobutyl-1-(1-phenylethyl)pyrrolidine-2,5-dione	259	259, 244, 216, 203, 188, 146, 132, 120, 106, 105, 104, 99, 79, 77
3-(1-Methylpentyl)-1-(1-phenylethyl)pyrrolidine-2,5-dione	287	287, 272, 244, 230, 203, 174, 160, 146, 132, 126, 120, 105, 104, 99, 79, 77, 55
3-Cyclopentyl-1-(1-phenylethyl)pyrrolidine-2,5-dione	271	271, 256, 228, 203, 174, 161, 160, 146, 132, 120, 105, 104, 99, 79, 77, 67, 55
3-Cyclohexyl-1-(1-phenylethyl)pyrrolidine-2,5-dione	285	285, 270, 242, 203, 174, 161, 160, 146, 132, 120, 105, 104, 99, 79, 77, 67, 55
3-(Cyclohexylmethyl)-1-(1-phenylethyl)pyrrolidine-2,5-dione	299	299, 256, 216, 203, 188, 174, 160, 146, 132, 120, 112, 105, 104, 99, 79, 77, 67, 55
3-Phenyl-1-(1-phenylethyl)pyrrolidine-2,5-dione	279	279, 265, 251, 236, 222, 160, 146, 132, 120, 105, 104, 91, 79, 77
3,4-Diphenyl-1-(1-phenylethyl)pyrrolidine-2,5-dione	355	355, 222, 181, 180, 179, 178, 165, 146, 132, 118, 105, 104, 91, 79, 77
3-Methyl-4-phenyl-1-(1-phenylethyl)pyrrolidine-2,5-dione	293	293, 291, 276, 263, 250, 246, 146, 132, 120, 119, 118, 117, 105, 104, 91, 79, 77
3-Phenyl-1-(1-phenylethyl)-3-propylpyrrolidine-2,5-dione	321	321, 279, 175, 146, 132, 131, 120, 118, 105, 104, 103, 91, 79, 77
3-Benzyl-1-(1-phenylethyl)pyrrolidine-2,5-dione	293	293, 250, 189, 174, 161, 160, 146, 132, 120, 117, 105, 104, 91, 79, 77, 65, 55
1,3-Bis-(1-phenylethyl)pyrrolidine-2,5-dione	307	307, 264, 203, 188, 174, 161, 161, 146, 132, 131, 120, 105, 104, 99, 91, 79, 77, 55
3-(1-Phenylbutyl)-1-(1-phenylethyl)pyrrolidine-2,5-dione	335	335, 203, 188, 174, 160, 146, 133, 132, 120, 117, 115, 105, 99, 91, 79, 77, 65, 55
3-(1-Methyl-4-phenylbutyl)-1-(1-phenylethyl)pyrrolidine-2,5-dione	349	349, 230, 203, 186, 174, 160, 146, 132, 126, 120, 105, 104, 99, 91, 79, 77, 65, 55
3-(4-Isopropylbenzyl)-1-(1-phenylethyl)pyrrolidine-2,5-dione	335	335, 292, 231, 230, 188, 174, 161, 160, 159, 146, 145, 133, 132, 120, 117, 105, 104, 91, 79, 77, 55
3-(2-Naphthylmethyl)-1-(1-phenylethyl)pyrrolidine-2,5-dione	343	343, 300, 239, 195, 174, 167, 154, 141, 128, 120, 115, 105, 79, 77, 55
3-(1-Phenylethyl)-3-azabicyclo[3.3.0]octane-2,4-dione	243	243, 200, 146, 132, 120, 105, 104, 103, 79, 77, 68
3-Hydroxy-1-(1-phenylethyl)pyrrolidine-2,5-dione	219	219, 201, 191, 185, 176, 132, 120, 105, 104, 79, 77
3-(1-Methylpropyl)-1-(1-phenylethyl)pyrrolidine-2,5-dione	259	259, 244, 216, 203, 174, 161, 160, 146, 132, 120, 113, 105, 104, 99, 79, 77, 69, 55
3-(1-Methylbutyl)-1-(1-phenylethyl)pyrrolidine-2,5-dione	273	273, 258, 230, 203, 174, 161, 160, 146, 132, 126, 120, 105, 104, 99, 79, 77, 69, 55
3-(1-Methylhexyl)-1-(1-phenylethyl)pyrrolidine-2,5-dione	301	301, 286, 258, 230, 203, 174, 161, 160, 146, 132, 126, 120, 105, 104, 99, 79, 77, 69, 55
3-(1-Methylheptyl)-1-(1-phenylethyl)pyrrolidine-2,5-dione	315	315, 300, 272, 230, 203, 174, 161, 160, 146, 132, 126, 120, 105, 104, 99, 79, 77, 69, 55
3-(1-Methyloctyl)-1-(1-phenylethyl)pyrrolidine-2,5-dione	329	329, 314, 286, 230, 203, 174, 161, 160, 146, 132, 126, 120, 105, 104, 99, 79, 77, 69, 55
3-(1-Methylnonyl)-1-(1-phenylethyl)pyrrolidine-2,5-dione	343	343, 328, 300, 230, 203, 174, 161, 160, 146, 132, 126, 120, 105, 104, 99, 55
3-(1-Methyldecyl)-1-(1-phenylethyl)pyrrolidine-2,5-dione	357	357, 314, 230, 203, 174, 160, 146, 132, 126, 120, 105, 99, 55
3-(Methylcyclopentyl)-1-(1-phenylethyl)pyrrolidine-2,5-dione	285	285, 270, 242, 203, 174, 161, 160, 146, 132, 120, 105, 104, 99, 79, 77, 67, 55
3-(2-Methylbenzyl)-1-(1-phenylethyl)pyrrolidine-2,5-dione	307	307, 264, 203, 174, 160, 146, 132, 131, 120, 117, 105, 104, 91, 79, 77, 55
3-(3-Methylbenzyl)-1-(1-phenylethyl)pyrrolidine-2,5-dione	307	307, 264, 203, 174, 160, 146, 132, 131, 120, 117, 105, 104, 91, 79, 77, 55
3-(4-Methylbenzyl)-1-(1-phenylethyl)pyrrolidine-2,5-dione	307	307, 264, 203, 174, 160, 146, 132, 131, 120, 117, 105, 104, 91, 79, 77, 55
3-(4-Ethylbenzyl)-1-(1-phenylethyl)pyrrolidine-2,5-dione	321	321, 278, 217, 174, 161, 160, 146, 145, 133, 120, 119, 117, 105, 91, 79, 77, 55
3-[1-(4-Methylphenyl)ethyl]-1-(1-phenylethyl)pyrrolidine-2,5-dione	321	321, 278, 217, 216, 203, 202, 174, 160, 145, 131, 120, 119, 117, 105, 91, 79, 77, 55
1-(1-Phenylethyl)-3-(4-propylbenzyl)pyrrolidine-2,5-dione	335	335, 292, 231, 230, 202, 188, 174, 161, 160, 159, 146, 145, 133, 132, 120, 117, 105, 104, 91, 79, 77, 55
3-[1-(4-Ethylphenyl)ethyl]-1-(1-phenylethyl)pyrrolidine-2,5-dione	335	335, 231, 230, 216, 174, 159, 146, 133, 120, 117, 105, 91, 79, 77, 55
1-(1-phenylethyl)-3-[1-(4-propylphenyl)ethyl]pyrrolidine-2,5-dione	349	349, 245, 244, 230, 174, 160, 159, 147, 131, 120, 117, 105, 91, 79, 77, 55
3-[1-(4-Isopropylphenyl)ethyl]-1-(1-phenylethyl)pyrrolidine-2,5-dione	349	349, 245, 231, 174, 160, 159, 147, 131, 120, 117, 105, 91, 79, 77, 55

*Identified by standard comparison
